# Role of dienelactone hydrolases in PET biodegradation by flavobacteria *Maribacter dokdonensis* and *Arenibacter palladensis*

**DOI:** 10.1128/aem.01698-25

**Published:** 2026-04-24

**Authors:** Ly T. T. Trinh, Colin Ochmann, Ifey Alio, Pablo Perez-Garcia, Sabine Keuter, Robert Dierkes, Lena Preuss, Christel Vollstedt, Wolfgang R. Streit

**Affiliations:** 1 Department of Microbiology and Biotechnology, University of Hamburg 14915, Hamburg, Germany; 2 Institute of Carbon Cycles, Helmholtz Zentrum Hereon, Geesthacht, Germany; Kyoto University, Kyoto, Japan

**Keywords:** Bacteroidota, Flavobacteria, *Maribacter dokdonensis*, *Arenibacter palladensis*, biofilms on plastics, PET degradation, polyethylene terephthalate (PET), PETase, micro- and nanoplastics

## Abstract

Dienelactone hydrolases (DLHs, EC 3.1.1.45) are bacterial enzymes primarily involved in the breakdown of cyclic esters. So far, this enzyme class is generally not well-known for PET degradation, and its potential role in plastic depolymerization remains largely unexplored. In the current study, we show that bacteria affiliated with the Bacteroidota (class Flavobacteria) harbor DLHs acting on PET foil and powder. We report on the isolation of two marine bacterial strains, *Arenibacter palladensis* UHH-Hm9b and *Maribacter dokdonensis* UHH-5R5, forming biofilms on PET foil and releasing µM amounts of terephthalic acid after 5–7 days. Genome sequencing and functional analyses identified two secreted DHLs designated PET93 and PET94 involved in PET degradation. While their predicted active sites and substrate-binding pockets were identical to previously published PETases, both enzymes differed largely in their structural features from known PETases and represent novel scaffolds. Further, they lacked the typical porC domain of the known PETases from the Flavobacteria. Biochemical characterization of the two recombinant enzymes confirmed activity on PET, the primary degradation products Bis(2-Hydroxyethyl) terephthalate (BHET) and Mono-(Hydroxyethyl) terephthalate (MHET). These are the first DLHs identified in the phylum of the Bacteroidota that are hydrolyzing plastics. Our findings indicate that Flavobacteria harbor an unexpectedly wide range of PET-active promiscuous enzymes.

**IMPORTANCE:**

Global plastics pollution is a major environmental challenge, and we still have limited knowledge of marine microbiota involved in possible remediation. Our research shows that marine Flavobacteria harbor the potential for PET degradation using dienelactone hydrolases (DLHs, EC 3.1.1.45). The widespread distribution of these microorganisms and the notion that these enzymes are secreted may imply a possible role in marine PET remediation.

## INTRODUCTION

Recent estimates indicate that at least 8–10 million tons of plastic waste are introduced into the ocean annually (1–4). While most of the plastic initially exists as larger floating fragments, exposure to weathering factors such as sunlight, wind, and water currents gradually breaks it down into smaller particles, from micro to nano sizes, which are smaller than 5 mm in diameter and more spreadable in air, water, and soil (5, 6). These particles, along with the chemicals they contain, are believed to negatively impact ecological systems and biodiversity across all environments (7, 8). Polyethylene terephthalate (PET), a widely used plastic polymer, has been accumulating in the oceans over the past decades, and we have only a limited understanding of the extent to which this polymer can be degraded by microorganisms. While PET is one of the commodity polymers that can be enzymatically degraded under laboratory and industrial-scale conditions, there is little knowledge of the microbiota potentially involved in marine PET degradation (9). Today, 311 microbial PET hydrolases (wildtype enzymes) have been described (for a complete list see PAZy database, www.pazy.eu; accessed on 7 February 2026) (10).

It is currently known that PET is in general degraded by promiscuous and secreted hydrolases affiliated with the esterase enzyme class (EC 3.1.1) They are either designated carboxylesterases (EC 3.1.1.1), poly(ethylene terephthalate) hydrolases (EC 3.1.1.101), lipases (EC 3.1.1.3), or cutinases (EC 3.1.1.74). All have a relatively wide substrate range and act mostly unspecifically on the polymer (11–13). Notably, among the currently known and functional PETases, no dienelactone hydrolases (DLHs) (3.1.1.45) are listed (see PAZy database [10]).

Recent research from our laboratory identified PET-degrading enzymes, PET27 and PET30, derived from the phylum of the marine Bacteroidota (14). The two enzymes were unique as they were secreted using the T9SS. Therefore, both enzymes harbored a porC and N-terminal secretion signal. While the overall turnover rates of the enzymes were rather low, they were active at low temperatures. The Bacteroidota phylum represents a major evolutionary lineage within the domain Bacteria and is one of the dominant components of the marine microbiomes. This phylum is particularly notable for its ability to degrade a wide range of natural polymers through multi-enzyme complexes, making them a valuable source of biologically active compounds (15). It is a highly diverse group of gram-negative bacteria that encompasses at least four distinct classes: Bacteroidia, Flavobacteria, Sphingobacteria, and Cytophagia (16). Among them, Flavobacteria is the largest and most widely distributed class of Bacteroidota, which are known for their surface-active properties and are frequently found associated with marine algae and sponges (17, 18). Flavobacteria are also one of the dominant classes on floating plastic particles such as LDPE, PP, and PET and major contributors to marine polysaccharide degradation (19–21).

In this study, we provide strong evidence that the flavobacterial isolates, *Maribacter dokdonensis* (UHH-5R5) and *Arenibacter palladensis* (UHH-Hm9b), code for active but promiscuous PET hydrolases, PET93 and PET94, enabling PET degradation while growing in biofilms on PET-foils. The two novel enzymes are the first DLHs (3.1.1.45) reported to be active on this polymer. Altogether, our findings indicate that the Bacteroidota harbor an unexpectedly wide range of PET-active enzymes potentially involved in polymer degradation in the marine environment.

## RESULTS

### Enriching for PET-active enzymes affiliated with the Bacteroidota

**Fig 1:**
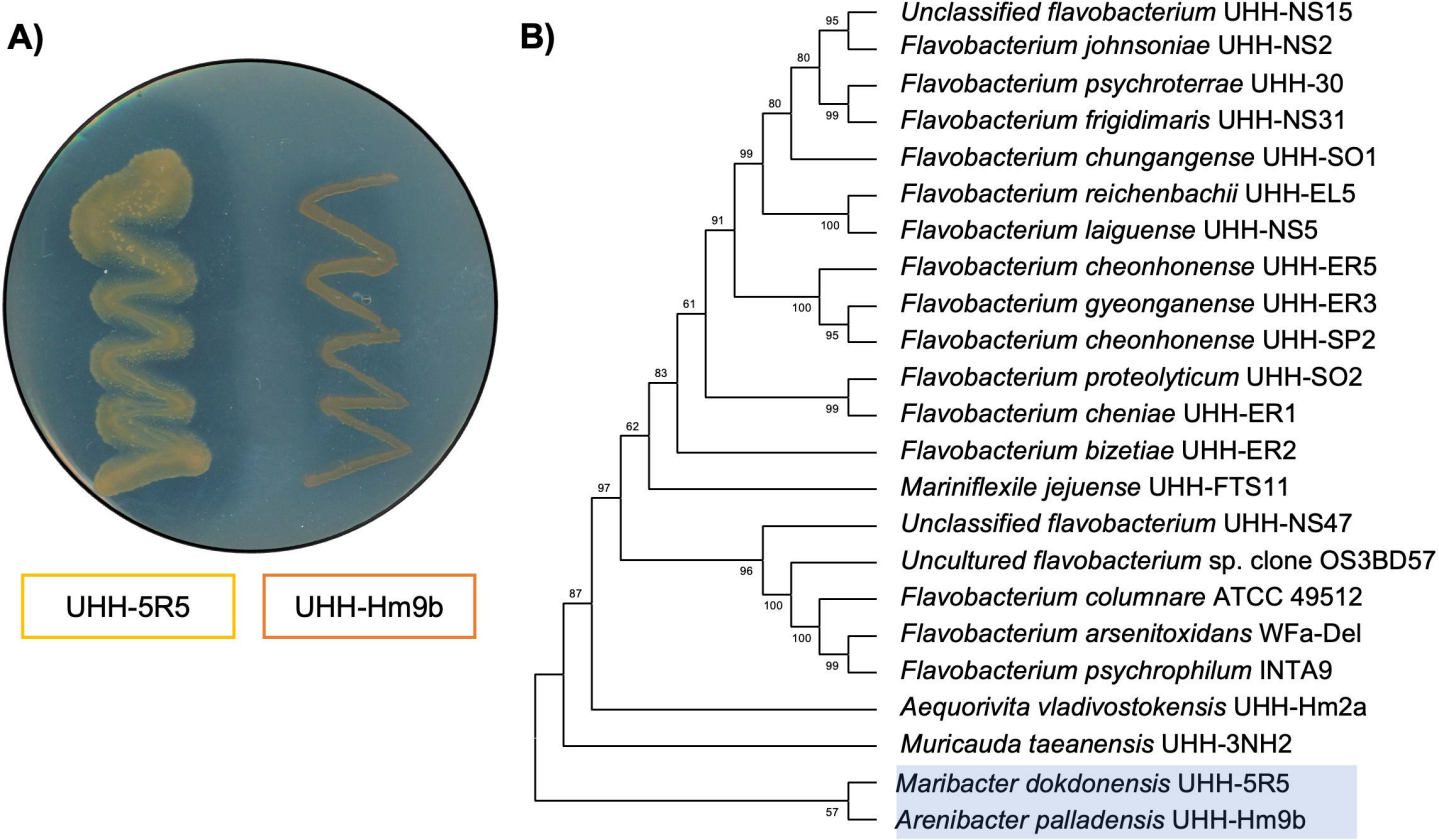
(**A**) Visualization of BHET-hydrolyzing activity by the isolates: *Maribacter dokdonensis* UHH-5R5 and *Arenibacter palladensis* UHH-Hm9b. Clear zones (halos) formed around bacterial colonies grown on BHET agar plates indicate enzymatic hydrolysis of the substrate. Plates were incubated at 28°C for 5 days. (**B**) Phylogenetic tree based on 16S rDNA gene sequences of the two newly isolated strains (highlighted in blue) and other members of the Bacteroidota. The tree was constructed using MEGAX, and the numbers displayed at nodes are the bootstrap values based on 1,000 replications.

To expand the functional diversity of PET-active enzymes within the phylum Bacteroidota, we enriched for aerobic Flavobacterial species from environmental samples using either BMB or R2A culture medium supplemented with PET powder as described in the Materials and Methods section. The different enrichment cultures resulted in a total of 19 distinct isolates as summarized in Table 1. Among them, four isolates were included that had been obtained in previous studies from our laboratory. The phylogenetic affiliation of the isolates was initially verified using 16S rDNA gene analysis. All isolates were affiliated with the phylum of the Bacteroidota, and the majority were affiliated with the class Flavobacteria. As a further initial screening to assess if any of these isolates indeed was able to secrete PET-active hydrolases (i.e., esterase E.C. 3.1.1.1), the isolates were tested for their capability to hydrolyze typical model substrates commonly used to screen for PET-active bacteria (e.g., TBT, PCL, and BHET) on agar plates. Halo formation was used as an indicator for the production and secretion of active hydrolases. Among all tested isolates, only two, UHH-5R5 and UHH-Hm9b, produced visible halos and were able to hydrolyze BHET, while only UHH-5R5 acted on both BHET and PCL (Fig. 1A). Using 16SrDNA analyses, we confirmed that UHH-5R5 and UHH-Hm9b phylogenetically cluster closely together and form a distinct branch within the Bacteroidota clade and belong to the class of Flavobacteria (Fig. 1B). Strain UHH-Hm9b is affiliated with the species *A. palladensis*, while the UHH-5R5 isolate is affiliated with the species *M. dokdonensis* (Table 1).

**TABLE 1:** Flavobacteriaceae isolates enriched in this work using PET powder as substrate

Isolate ID	Affiliated species (based on 16S rDNA sequencing and at >99% identity)^*a*^	IMG accession	Origin
UHH-Hm2a	Aequorivita vladivostokensis*	298716	Marine Aquaculture, Büsum, Germany
UHH-Hm9b	Arenibacter palladensis*	294450	Marine Aquaculture, Büsum, Germany
UHH-3NH2	Muricauda taeanensis*	298717	Marine Aquaculture, Büsum, Germany
UHH-5R5	Maribacter dokdonensis*	294449	Marine Aquaculture, Büsum Germany
UHH-ER1	Flavobacterium cheniae*	341742	North Sea, Hamburg, Germany
UHH-ER2	Flavobacterium bizetiae*	298720	Elbe River Sediment, Hamburg, Germany
UHH-ER3	Flavobacterium gyeonganense*	341743	Elbe River Sediment, Hamburg, Germany
UHH-SO1	Flavobacterium chungangense*	341744	Terrestrial Isolate, Hamburg, Germany
UHH-SO2	Flavobacterium proteolyticum*	298719	Terrestrial Isolate, Hamburg, Germany
UHH-ER5	*Flavobacterium cheonhonense*	NA^*b*^	Elbe River Sediment, Hamburg, Germany
UHH-EL5	*Flavobacterium reichenbachii*	NA	Lake “Außenalster,” Hamburg, Germany
UHH-SP2	*Flavobacterium cheonhonense*	NA	Lake Stadtpark, Hamburg, Germany
UHH-NS2	*Flavobacterium johnsoniae*	NA	North Sea, Hamburg, Germany
UHH-NS5	*Flavobacterium laiguense*	NA	North Sea, Hamburg, Germany
UHH-NS15	Unclassified flavobacterium	NA	North Sea, Hamburg, Germany
UHH-NS30	Flavobacterium psychroterrae	NA	North Sea, Hamburg, Germany
UHH-NS31	Flavobacterium frigidimaris	NA	North Sea, Hamburg, Germany
UHH-NS47	Unclassified flavobacterium	NA	North Sea, Hamburg, Germany
UHH-FTS11	*Mariniflexile jejuense*	NA	Marine Aquaculture, Büsum

^*a*^ Sequenced genomes are marked with an asterisk (*).

^*b*^ NA, not available.

### *M. dokdonensis* UHH-5R5 and *A. palladensis* UHH-Hm9b form dense biofilms on PET foil and release TPA

Intrigued by the above-made observations, we asked if both isolates would grow on PET foil, form biofilms, and release small amounts of TPA (terephthalic acid) as one of the primary PET degradation products. To assess if UHH-5R5 and UHH-Hm9b degrade PET foil under biofilm conditions, we inoculated both wild-type strains in BMB medium with PET foil as a possible substrate and surface to form biofilms on. The attachment and biofilm formation of UHH-5R5 and UHH-Hm9b on PET were observed over a 7-day incubation period using laser scanning microscopy and potential PET degradation was analyzed using UHPLC to detect the released TPA.

**Fig 2:**
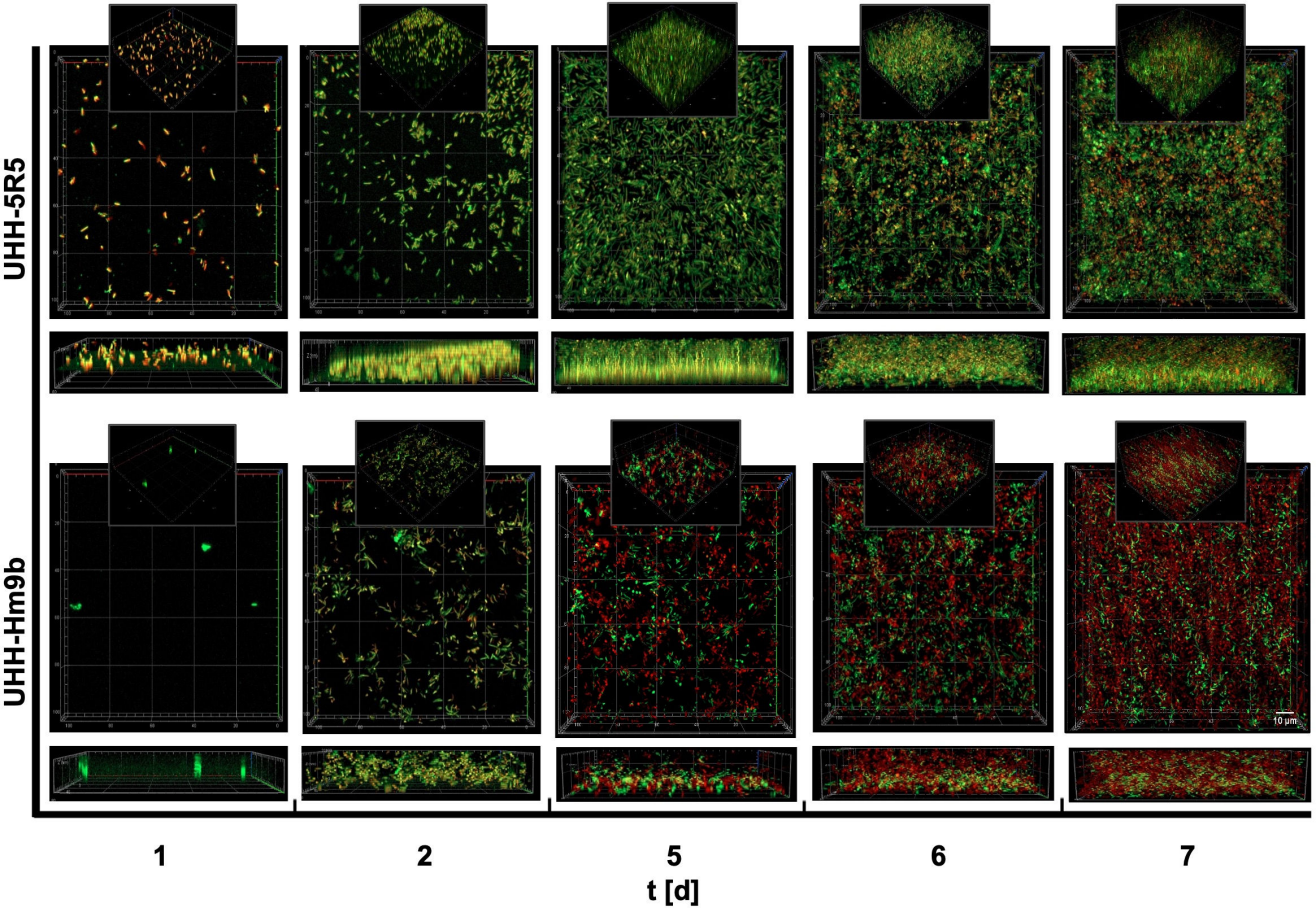
Fluorescence microscopy images showing the development of biofilms formed by the Bacteroidota isolates UHH-5R5 and UHH-Hm9b on PET foil over a 7-day incubation period. Biofilms were visualized using Live/Dead staining, where live cells fluoresce green (SYTO 9) and dead cells fluoresce red (Propidium iodide). Images were captured on days 1, 2, 5, 6, and 7 using a confocal laser scanning microscope (CLSM) with a 63× oil immersion objective. For each sample, at least three different positions were observed, and all images here are representative of three independent biological replicates.

Initially, only a few individual cells of both strains were sparsely adhered to the PET surface, with no visible biofilm structures. However, on day 2, thin biofilm layers formed, accompanied by an increased number of attached cells (Fig. 2). The 2-day-old biofilms of UHH-5R5 and UHH-Hm9b exhibited an average thickness of 5.9 ± 1.04 and 8.4 ± 1.14 µm, respectively, corresponding to approximately three to five cell layers on foils. An increase in biofilm development was observed at day 3, with biofilm thickness reaching 6.7 ± 2.1 µm (UHH-5R5) and 12 ± 3.2 µm (UHH-Hm9b). At day 6, both strains developed well-structured, multilayered biofilms, reaching thicknesses of 14.5 ± 1.8 µm (UHH-5R5) and 13.4 ± 1.2 µm (UHH-Hm9b). Interestingly, while the biofilm thickness of UHH-5R5 decreased slightly to 11.3 ± 2.5 µm by day 7, that of UHH-Hm9b remained stable at 15.1 ± 3.1 µm (Table S1).

**Fig 3:**
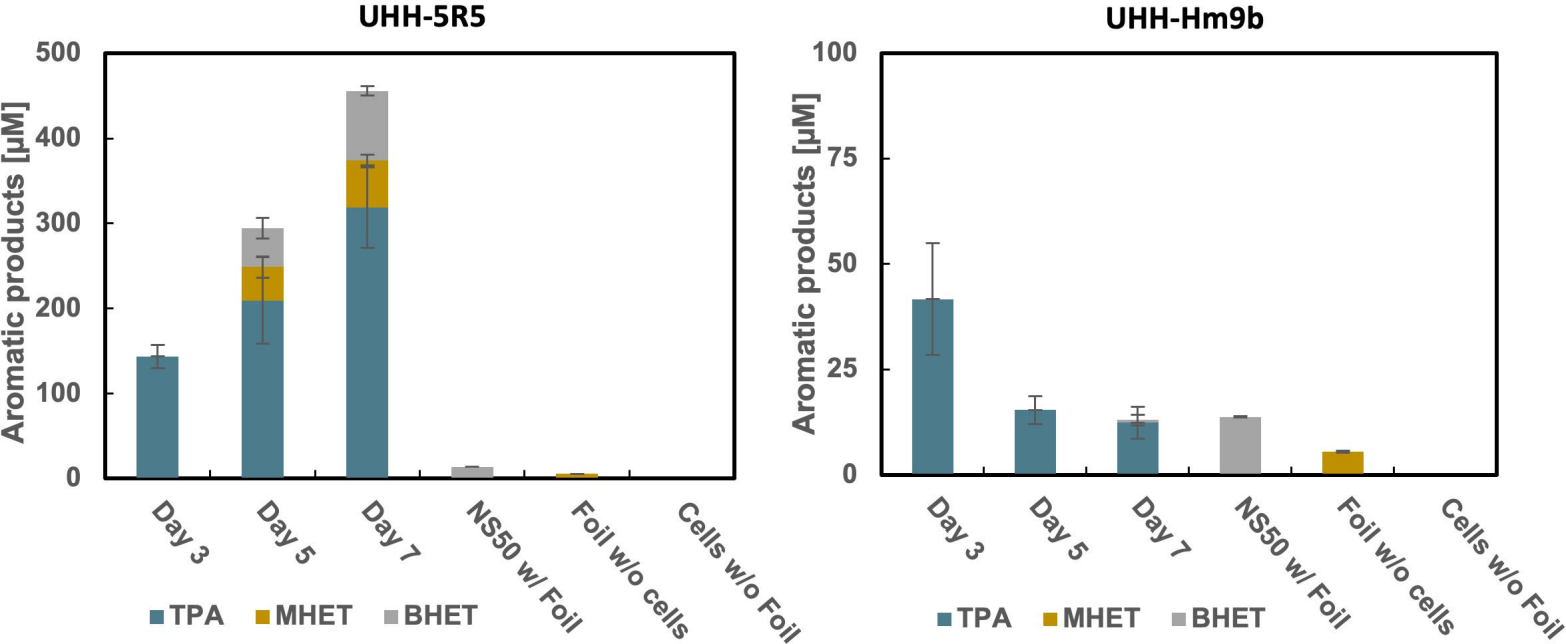
PET degradation products observed in supernatants from UHH-5R5 and UHH-Hm9b biofilms grown on PET film. Supernatants were collected after 3, 5, and 7 days, and the degradation products were quantified using UHPLC as detailed in the Materials and Methods section. Negative control samples were taken after 7 days of incubation. Data are normalized and corrected relative to *Escherichia coli* DH5α growing on PET foil. PET degradation products in supernatants from UHH-5R5 and UHH-Hm9b biofilms grown on PET film. Values are the mean of three biologically independent replicates, with error bars representing standard deviations.

To detect possible PET degradation products, we collected and concentrated supernatants from 3-, 5-, and 7-day-old biofilms grown on PET foil. UHPLC analysis revealed that strain UHH-5R5 released approximately 100–320 µM of TPA in the concentrated supernatants, equivalent to approximately 6–19 µM in the original samples (corresponds to 60–190 nmol in 10 mL culture volume), with smaller amounts of MHET and BHET detected throughout the observation period (Fig. 3). The concentration of TPA steadily increased from day 3 to day 7, reaching a maximum of approximately 318 ± 47.2 µM on day 7, indicating a slow but continuous degradation process over time. In contrast, strain UHH-Hm9b exhibited lower overall PET degradation activity. Nevertheless, UHPLC analysis confirmed its ability to release TPA from biofilms on PET foil at micromolar level. Interestingly, TPA concentration was highest on day 3 (41.6 ± 13.2 µM in concentrated samples or 2.5 ± 0.8 µM in original samples, corresponds to 25 ± 8 nmol in 10 mL culture volume) but subsequently decreased to approximately 12 ± 3.8 µM (7.2 ± 2.2 nmol in 10 mL culture volume) on day 7 (Fig. 3). Altogether, these findings imply that UHH-5R5 and UHH-Hm9b form dense biofilms on non-treated PET foil and can release minute amounts of TPA under the tested conditions in the laboratory.

### Genome sequencing identifies two novel dienelactone hydrolases involved in PET degradation

**Fig 4:**
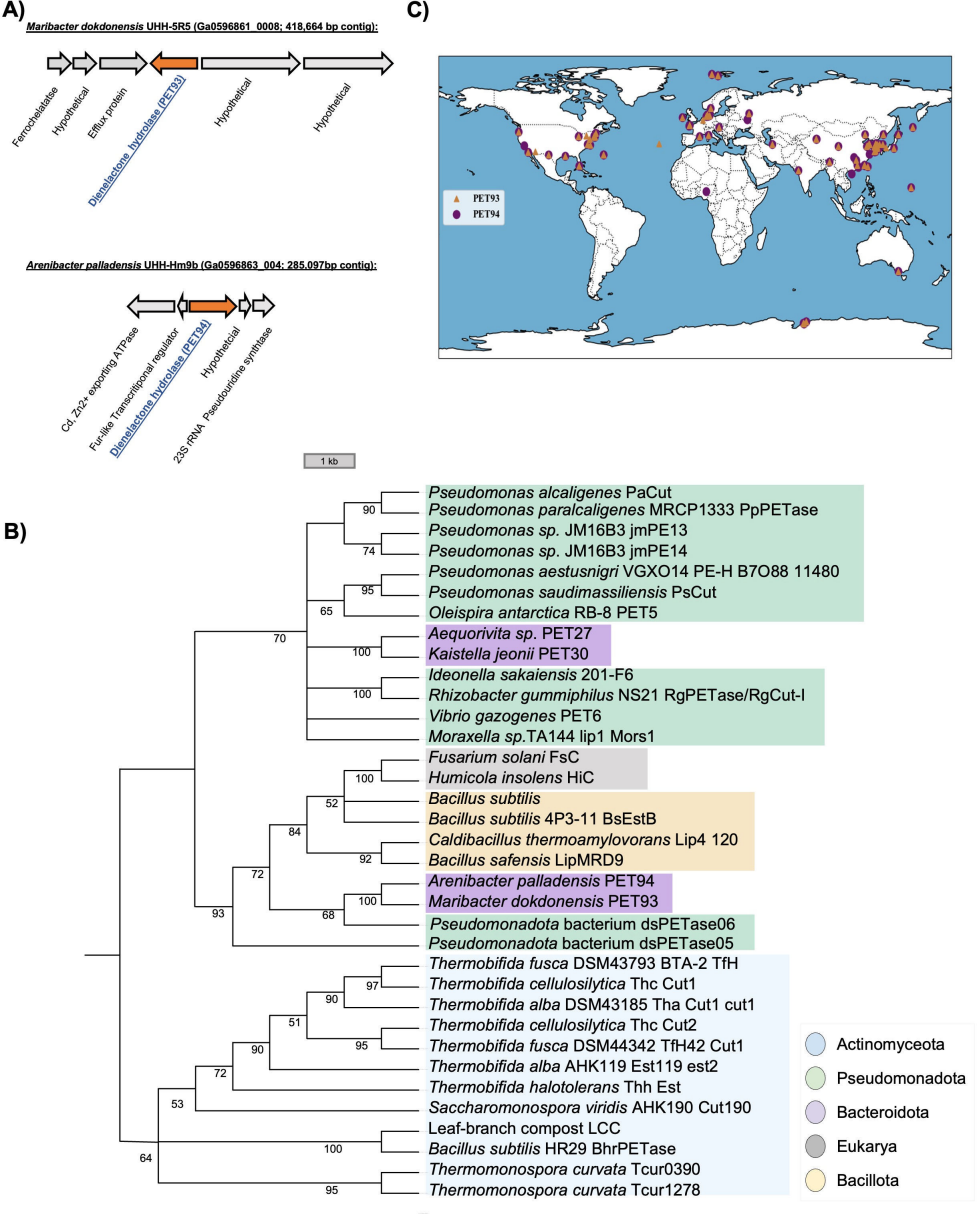
(**A**) Genetic context of the dienelactone hydrolases PET93 and PET94 located in the Bacteroidota strains *Maribacter dokdonensis* UHH-5R5 and *Arenibacter palladensis* UHH-Hm9b, respectively. (**B**) Neighbor-joining trees showing the phylogenetic relationship of PET93 and PET94 with other well-characterized enzymes. The tree was constructed using MEGAX and the number displayed at nodes is the bootstrap values based on 1,000 replications. (**C**) Global distribution of PET93 and PET94 homologs. The corresponding contigs containing the two hydrolases are available for download from IMG using the indicated accession numbers. Enzyme entries were retrieved from the PAZy database.

To identify the possible genes and enzymes linked to the observed hydrolytic activities, the genome of both isolates was sequenced. The draft genomes of both isolates were deposited at IMG under accession numbers 294449 and 294450. The genome assemblies implied a size of 10.2 Mbp for *M. dokdonensis* UHH-5R5 and 6.1 Mbps for *A. palladensis* UHH-Hm9b. A detailed analysis of both draft genomes and using the previously published HMM search motifs (22) for PETases revealed a single hit (*e* value < 1e−5) for possible PETases in each strain. The potential PET-acting esterase in Arenibacter corresponded to the predicted ORFs (Genome ID 8000214887/Locus Tag Ga0596863_004_127947_129248/Gene ID 8000215898) and in Maribacter (Genome ID 8000203477/Locus Tag Ga0596861_0008_300124_301428/Gene ID 8000209034) was identified as potential PETase (Fig. 4A). The potential PET-esterase predicted in the two isolates was designated PET93 and PET94, respectively. Both hydrolase coding genes were not part of conserved regions on the bacterial chromosomes, and only a few other strains were observed with similar gene neighborhoods in the genomes available in IMG. None of the predicted genes were part of an operon, and the flanking genes appeared to be transcribed in the opposite direction. For both predicted genes, putative promoter sequences were identified, with an SD sequence 5–6 bp upstream of the translational start point.

**Fig 5:**
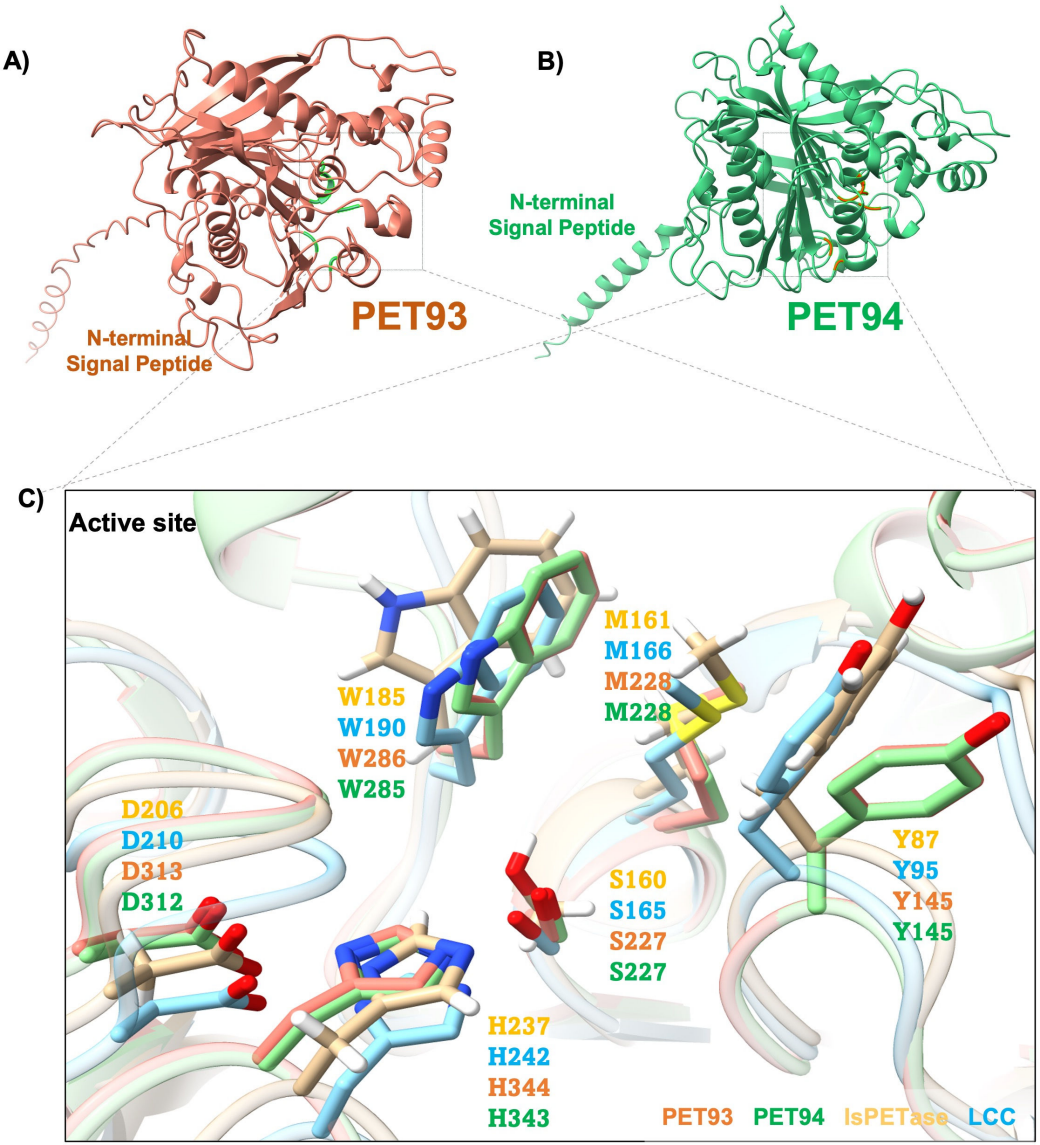
Structural model of PET-hydrolyzing enzymes from Bacteroidota. (**A**) Overall structure of PET93, including the active site and potential substrate-binding site. (**B**) Overall structure model of PET94, including the active site and potential substrate-binding site. (**C**) Comparison of active site residues. All four enzymes PET93 (orange), PET94 (green), *Is*PETase (yellow, PDB 6EQE), and LCC (light blue, PDB 4EBO) have the typical residues of Ser-hydrolases at the catalytically active positions (Ser, His, and Asp) and also have the same amino acids associated with PET-binding. The residues of *Is*PETase and LCC are indicated in light orange and light blue, respectively.

The predicted esterase PET93 from *M. dokdonensis* consisted of 434 amino acids with a possible secretion signal cleaving site identified between positions 27 and 28 (VNA-QT) using the Sec/SPI secretion system. Similarly, the predicted protein PET94 derived from *A. palladensis* consisted of 433 amino acids and an N-terminal cleavage site between positions 25 and 26 (LNA-QT). Both amino acid sequences of the enzymes were highly similar (>99%) to predicted DLHs in closely related species either affiliated with the genera Maribacter (PET93) or Arenibacter (PET94) in the NCBI database. However, their overall amino acid similarity to known PETases from the phylum of the Bacteroidota was relatively low, with less than 50% identity (Table 2). Structural analysis also revealed a low similarity between the *Ideonella sakaiensis (Is*) PETase (*Is*PETase, PDB: 6EQE) structure and modeled structures of PET93 and PET94. Notably, the predicted active sites and binding sites of PET93 and PET94 were identical in their amino acid sequence to those of *Is*PETase and the leaf-branch compost cutinase (LCC, PDB: 4EBO) (Fig. 5; Table 2).

**TABLE 2:** Sequence similarities generated for PET93 and PET94 against functionally verified PET hydrolases^*a*^

Enzyme	Origin	Sequence identity (%)		Active site	Binding site
		PET93	PET94		
*Is*PETase	*Ideonella sakaiensis*	49.5	48.6	Ser Asp His	Tyr Met Trp
PET27	*Aequorivita* sp.	50	48.6	Ser Asp His	Phe Met Trp
PET30	*Kaistella jeonii*	47.9	46.2	Ser Asp His	Phe Met Trp
LCC	Leaf compost metagenome	48.5	49.4	Ser Asp His	Tyr Met Trp

^*a*^ Active site and PET-binding motifs of PET93 and PET94 with selected and known PETases. For PET93 and PET94, the predicted active site motif is Ser Asp His, and the binding site motif is Tyr Met Trp.

To evaluate the evolutionary relationships among bacteroidetal PET-hydrolyzing enzymes, including PET93, PET94, and other known PETases, a phylogenetic analysis was conducted using published and functionally verified PETases (Fig. 4B). The resulting consensus tree revealed that putative Bacteroidota PET-degrading hydrolases formed a distinct subcluster. Interestingly, PET93 and PET94 were not grouped within the subcluster that included the previously known Bacteroidotal enzymes PET27 and PET30. They also differed with respect to the secretion signal. While PET93 and PET94 are secreted enzymes and code for a predicted N-terminal secretion signal, no PorC-like C-terminal secretion signal was observed as it had been described for PET27 and PET30 previously.

### Proteomic analysis identified general expression of PET93 and PET94

To assess whether PET93 and PET94 were expressed at significant levels, strains UHH-5R5 and UHH-Hm9b were cultivated under planktonic and biofilm conditions in the presence or absence of BHET (1 mM) and/or PET foil. Samples were analyzed by quantitative proteomics in duplicates and combined for analysis. On average, we detected for both strains between 2,400 and 2,800 expressed proteins under the conditions tested (Table S4). When we ranked the most abundant proteins, we observed that in strain UHH-5R5 (Protein ID: NKFFJOOI_00264), it was detected at positions 428 and 429 in the planktonic-grown cell extracts (Table 3). In planktonic-grown cell extracts of UHH-Hm9b, PET94 (Protein ID: ICNPBAOJ_00708) was observed at position 1432 in the presence of BHET and position 1239 in the absence of BHET. In the biofilm-grown cell extracts, PET93 was detected at positions 309 and 1432, and PET94 was detected at position 862. Interestingly, we did not detect PET94 at biofilm conditions using PET foils (Table 3).

**TABLE 3:** Proteomic analyses and relative expression level of PET93 and PET94 in their host strains under planktonic and biofilm conditions^*a*^

Growth condition	Position in overall proteome data set	
	*M. dokdonensis* UHH-5R5 PET93^*b*^	*A. palladensis* UHH-Hm9b PET94^*c*^
Biofilm nothing added	309	862
Biofilm with PET foil	1432	ND^*d*^
Planktonic nothing added	428	1432
Planktonic with BHET (1 mM)	429	1239

^*a*^ Original data are available in Table S4.

^*b*^ PET93 (Protein ID: NKFFJOOI_00264).

^*c*^ PET94 (Protein ID: ICNPBAOJ_00708).

^*d*^ ND, not detected.

In UHH-5R5, PET93 (Protein ID: NKFFJOOI_00264) was identified with 142 peptide-spectrum matches (PSMs), 21 unique peptides, and 55% sequence coverage. BHET-grown cultures yielded 115 PSMs, 20 unique peptides, and 54% sequence coverage, while planktonic control showed 117 PSMs, 19 unique peptides, and 50% coverage. Together, these data indicate robust and reproducible detection of PET93 across growth conditions and substrates, with the highest evidence in biofilm control and planktonic BHET conditions.

In strain UHH-Hm9b, PET94 (Protein ID: ICNPBAOJ_00708) was reliably identified in most conditions but showed condition-dependent variability. Under planktonic conditions, PET94 was identified with low but still confident evidence. BHET-grown cultures yielded 23 PSMs, 9 unique peptides, and 26% coverage, while planktonic controls showed 30 PSMs, 9 unique peptides, and 25% coverage. In biofilm-grown samples, the protein was detected with 117 PSM, 19 unique peptides, and 50% sequenced coverage.

Altogether, these results imply stable expression in planktonic conditions with reduced detection under substrate-associated biofilm conditions.

### Partial biochemical characterization of potential PET93 and PET94

**Fig 6:**
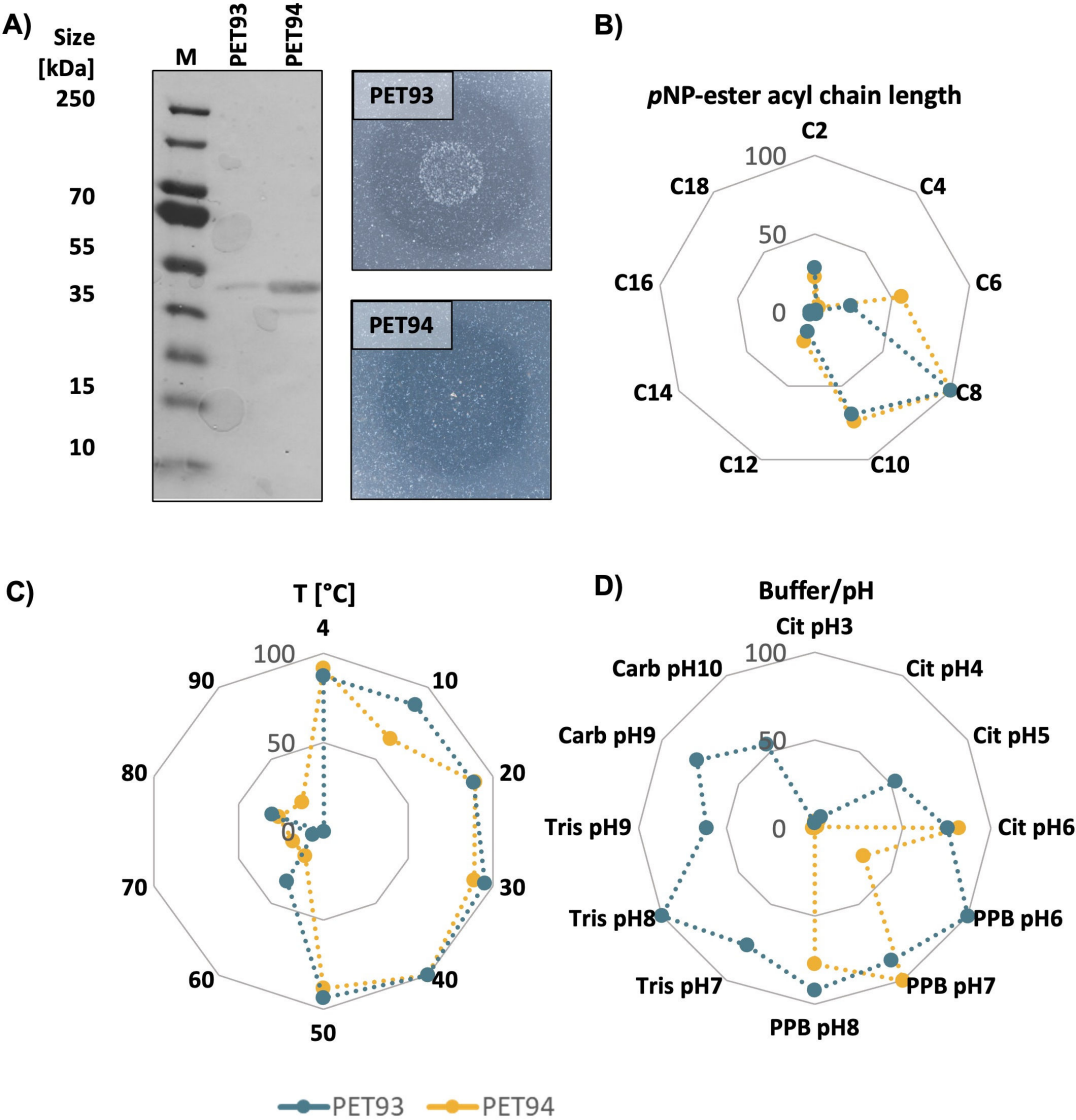
Purification and biochemical characterization of the dienelactone hydrolases PET93 and PET94. Purification of the His_6_-tagged dienelactone hydrolase PET93 (48.1 kDa) and PET94 (45.6 kDa) from *Maribacter dokdonensis* UHH-5R5 and *Arenibacter palladensis* UHH-Hm9b*,* respectively, and protein activity on BHET-containing plates (**A**). Substrate preference was tested with *p*NP-acetate (C2) to *p*NP-stearate (C18) (**B**). Temperature (**C**) and pH and buffer optimum (**D**) were tested with *p*NP-octanoate C8. The assays were conducted using 0.1 M buffers at pH 3–10 (pH 3–6, citrate buffer [Cit]; pH 6–8, potassium phosphate buffer [PPB]; pH 7–9, Tris buffer; pH 9–10, carbonate-bicarbonate buffer [Carb]). All assays were carried out in triplicate at 37°C for both enzymes. Data represent the mean values of three independent replicates, with standard deviation (SD) ≤10% for all measurements.

To further characterize both proteins and finally verify their PET-degrading activities, the gene sequences obtained by HMM were amplified from the corresponding organisms, cloned into *Escherichia coli* DH5α, *and* overexpressed in *E. coli* BL21(DE3). The transformed cells produced 48.1 and 45.6 kDa proteins when induced with 1 mM IPTG. For both the C-terminal 6x histidine-tagged proteins, we obtained recombinant proteins using Ni-NTA purification protocols with relatively high purity and activity on BHET plates (Fig. 6A). We first characterized the activity of the two recombinant enzymes using the *p*NP assay. A substrate spectrum was recorded with *p*NP-esters, which have acyl chain lengths of 4–18 carbon atoms. PET93 and PET94 revealed a narrow spectrum of substrates they hydrolyzed. The substrate specificity tests clearly showed that PET93 and PET94 can hydrolyze ester bonds of a chain length of 6–10 carbon atoms but exhibit highest activity to hydrolyze a chain length of C8 *p*NP-octanoate (Fig. 6B). The hydrolyzing activity of both enzymes was also assessed on the temperature range from 4°C to 90°C. Both enzymes were shown to perform best at 40°C, and interestingly, they were able to act at 4°C (Fig. 6C), implying a possible cold adaptation.

Buffers with pH between 3 and 10 were used to discover the optimal pH conditions for PET93 and PET94. With C8 *p*NP substrate, it was shown that PET93 was most active in 0.1M citrate buffer pH 6 and in 0.1 M potassium phosphate buffer pH 7, while PET94 was highly active in potassium phosphate buffer pH 6–8. PET93 lost its activity nearly completely in the citrate buffer at pH below 5, Tris buffer at pH values 7–9, and Carb buffer at pH values 9–10 (Fig. 6D). Further, it is shown that PET94 was almost inactivated under acidic conditions pH < 4.

### PET93 and PET94 activity toward MHET, BHET, and PET

Given that PET93 and PET94 are most active at 30–40°C in pH 7 potassium phosphate buffer, the recombinant enzymes were initially assayed with substrates MHET (1 mM) and BHET (5 mM). Therefore, UHPLC analysis was performed to identify the PET degradation product TPA (Table 4). Both enzymes can hydrolyze BHET and MHET to TPA and 0.2 mg mL^−1^ of PET93 released an average of 2,642.8 ± 46.9 µM TPA from BHET and 776.3 ± 29.5 µM TPA from MHET after 24 h in 200 µL reaction volume. Under the same conditions, PET94 (0.2 mg mL^−1^) also released a similar amount of 2,659.8 ± 99.6 and 684.5 ± 49.1 µM TPA when incubated with BHET and MHET, respectively.

**TABLE 4:** Recombinant and purified PET93 and PET94 enzymatic hydrolysis of MHET [mono(2-hydroxyethyl) terephthalate], BHET [bis(2-hydroxyethyl) terephthalate], and PET (polyethylene terephthalate) with their final degradation product TPA (terephthalic acid)^*a*^

Substrate added	Treatment	BHET detected (µM), mean ± SD	MHET detected (µM), mean ± SD	TPA detected (µM), mean ± SD
BHET	Control	2,244 ± 38.4	286.6 ± 11.5	ND^*b*^
	PET93	ND	ND	2,642.8 ± 46.9
	PET94	ND	ND	2,659.8 ± 99.6
MHET	Control	ND	736 ± 31.5	20.1 ± 0.73
	PET93	ND	ND	776.3 ± 29.5
	PET94	ND	ND	684.5 ± 49.1
PET foil*	Control	ND	ND	3.17 ± 1.35
	PET93	ND	ND	26.63 ± 1.76
	PET94	ND	ND	37.3 ± 11.5
PET powder*	Control	ND	ND	3.31 ± 2.21
	PET93	ND	ND	37.06 ± 18.5
	PET94	ND	ND	51.39 ± 21.9

^*a*^ The concentration of the products was determined by UHPCL. The samples were incubated at 37°C with continuous shaking at 200 rpm and were analyzed in triplicates. BHET and MHET were added at 5 mM and 1 mM concentrations, respectively. *, PET substrates were pretreated with UV light for 1 week as indicated in Materials and Methods.

^*b*^ ND, not detected.

Notably, when non-treated PET foil or powder was used as substrate for these two enzymes at pH 7 and 37°C, no TPA was detected by UHPLC after 5 days of incubation. However, both enzymes were able to degrade UV-treated PET. After 5 days of incubation with UV-treated PET powder, reasonable levels of PET degradation product were observed in a 200 μL reaction volume by UHPLC analyses, reaching 40.4 ± 18.67 and 54.7 ± 21.9 µM TPA released by PET93 and PET94 (corresponding to 8.08 ± 3.73 and 10.94 ± 4.38 nmol), respectively. However, only about 29.8 ± 1.76 and 37.3 ± 11.5 µM TPA by PET93 and PET94 (corresponding to 5.96 ± 0.35 and 7.46 ± 2.30 nmol) were detected under the same conditions with UV-treated foil (Table 4). This suggests that UV probably helps promote the initial breakdown of PET.

**Fig 7:**
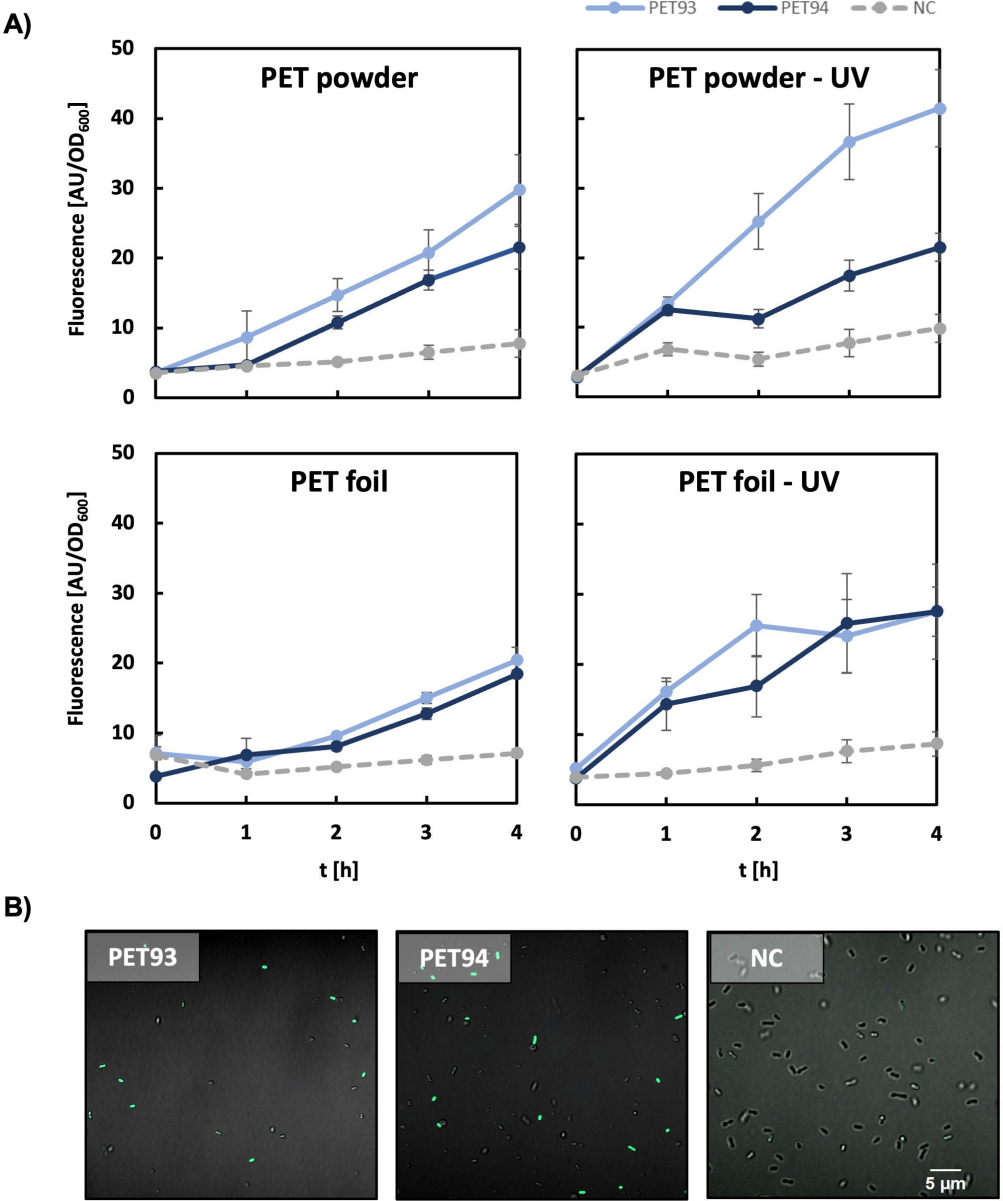
Detection of enzymatic TPA release from untreated and UV-treated PET powders and foils by *Comamonas thiooxidans* UHH04. Fluorescence response of *C. thiooxidans* UHH04 reporter cells (24) to supernatants from incubations of PET93 and PET94 with PET powder and foil, both untreated and UV-treated (**A**). Respectively, 0.1 mg mL^−1^ of each enzyme and BSA as a negative control (NC) were incubated with PET for 5 days in 0.1 M potassium phosphate buffer pH 7 before the addition of the UHH04 reporter to the supernatant. Fluorescence signals of sfGFP were normalized to the absorbance of the reporter cells at 600 nm. Data points represent mean values of six measurements, with standard deviation indicated by error bars. CLSM images of UHH04 reporter cells incubated with supernatants from the enzymatic reactions of PET93, PET94, and BSA (NC) on PET (**B**). The sfGFP fluorescence channel intensity was uniformly set for all images to allow for comparison.

Since we observed that the recombinant proteins exhibited relatively low turnover rates in the µM range, we used the recently published *Comamonas thiooxidans* S23 reporter strains (23) which can detect nM concentrations of TPA in further tests. This biosensor was used not merely to take advantage of its higher sensitivity, but rather to be used as an independent confirmation to exclude potential false positives. Using this reporter strain (ReporTPA_UHH04, Table S5)*,* we detected TPA release from PET foil incubated with recombinant PET93 and PET94 (Fig. 7A), whereas BSA controls showed no fluorescence (Fig. 7B). Altogether, the data implied that the recombinant enzymes PET93 and PET94 are both active on PET, albeit at relatively low levels.

### Global distribution of PET93 and PET94

We further analyzed the global distribution of PET93 and PET94 and their homologs. It is notable that we were able to identify homologs in more than 250 currently available genome sequences of other closely related strains from publicly available databases IMG/MER (threshold of 50% sequence identity and over 80% coverage) (25, 26). Analysis of their occurrence and frequency of PET93 and PET94 raised the question of the extent to which these enzymes could impact plastic degradation in the natural environment. For a focused view, we selected 99 homologs assigned to the Bacteroidota phylum in our global search to construct a global distribution map. Interestingly, these homologs originated from a broad range of countries and regions (Table S6), suggesting that the Bacteroideotal-derived enzymes PET93 and PET94 are widespread and may play a significant role in the global degradation of PET in nature (Fig. 4C).

## DISCUSSION

Recent studies showed that bacteria affiliated with the genera Maribacter and Arenibacter within the phylum of the Bacteroidota are globally distributed and play a significant role in the marine carbon cycle (27). They commonly inhabit marine sediments, coastal environments, and algae-associated habitats (28–33). Both genera demonstrate a broad and efficient capacity to degrade complex polysaccharides, playing a crucial role in organic matter cycling and microbial interactions in marine ecosystems (34–37). Notably, the occurrence of microbial species affiliated with both genera in the plastisphere has been described recently (38, 39). While it is well-known that bacteria in the plastisphere usually form biofilms on micro- or nanoparticles, it is commonly accepted that they mostly do not degrade the polymer (5, 40–42). However, it is assumed that they do primarily feed on the additives contained in the polymers and/or simply use the surface to attach and form biofilms (5, 14, 43).

While microorganisms in the phylum of the Bacteroidota are well known for their very diverse set of enzymes involved in polysaccharide hydrolysis, only a few species have been linked to plastic (i.e., PET) degradation to date (24). An initial HMM-based screen of metagenomic data sets has revealed that the Bacteroidota harbor considerable potential as yet underexplored sources of PET-degrading enzymes, particularly in the marine environment (22). The newly discovered enzymes PET93 and PET94 in this study are both promiscuous hydrolases (E.C. 3.1.1.45). While the native substrate of both enzymes remains currently unknown, our data showed a significant but slow turnover of PET (Fig. 3 and 7). Both enzymes differed largely in their structure from the previously published T9SS-dependent esterases involved in PET degradation. In fact, they represent novel scaffolds and imply that the phylum of the Bacteroidota harbors a diverse set of promiscuous enzymes to degrade these man-made polymers.

Notably, homologs of PET93 and PET94 were found globally in genome data sets covering a wide range of climate zones (Fig. 4C). It is likely due to the ability of Bacteroidota to decompose a wide range of biopolymers, including cellulose, algal polysaccharide (e.g. laminarin, alginate, and xylan), and other complex carbohydrates (44–46). Similar to other Bacteroidotal enzymes PET27 and PET30 (24), our enzymes demonstrated PET-hydrolyzing activity at temperature as low as 4°C, suggesting a potential role in slow, long-term degradation of PET microparticles in cold environments. While these data do not prove that PET93 and 94 are active in nature, it is likely that they are secreted and will be involved in enzymatic PET degradation. Recently, we showed that in *Vibrio gazogenes*, a PETase (PET6) was expressed constitutively at a low but significant level under various growth conditions (47). Assuming that in Maribacter and Arenibacter, similar regulatory pathways may exist, it is likely that PET93 and 94 are also expressed at low levels in their ecological niche.

Using our well-established HMM motif, we did not detect additional candidate genes associated with known PETase families, except PET93 and PET94. We, however, identified more than 40 potential lipases and esterases in both genomes (Tables S2 and S3). Some of these are potentially secreted as they contain signal peptides. Since esterases and lipases are, in general, promiscuous enzymes, we cannot exclude that some of the secreted and additionally identified esterases in the two bacteria are active on PET as well (Tables S2 and S3). Within these settings, the proteome data obtained strongly imply that PET93 and PET94 are secreted under planktonic conditions and that at least PET93 is significantly expressed in the laboratory under biofilm conditions as well (Table 3).

Interestingly, BHET and MHET accumulate in low concentrations in culture supernatants of *M. dokdonensis* UHH-5R5 and are not completely turned over. The accumulation of both PET degradation products in the culture supernatant of *M. dokdonensis* UHH-5R5 may reflect the limitations at the cellular level rather than intrinsic catalytic capacity of PET93 observed *in vitro*. PET93 expression levels and/or secretion efficiency *in vivo* may be low, resulting in insufficient extracellular enzyme concentration to fully process BHET and MHET to TPA. Further, *in vivo* conditions (e.g. pH, temperature, substrate accessibility, or the presence of inhibitory compounds) may reduce PET93 activity compared to optimized recombinant assays. Additionally, limited enzyme-substrate contact at the cell surface may also further constrain complete PET conversion to TPA. While our data do not allow us to claim the metabolic utilization of PET-derived compounds in the marine environment, the study delivers first evidence that in *M. dokdonensis* UHH-5R5 and *A. palladensis* UHH-Hm9b code for active dienelactone hydrolases that are expressed under laboratory conditions in their host and that both hydrolyze PET. Although the catalytic efficiency of PET93 and PET94 toward PET does not meet the level of industrial applications, their ecological significance should not be underestimated as they are derived from globally occurring microorganisms. Their global presence highlights that these microbes may play an important but not yet fully understood role in natural bioremediation of marine plastic pollution. Thus, future work will have to explore the role of the secreted DLHs PET93 and PET94 in their native environment.

## MATERIALS AND METHODS

### Enrichment, isolation, and identification of Bacteroidota strains

Microbial communities from marine and aquatic environments were enriched in 100 mL R2A or BMB media with 1 g of PET powder. Enrichment cultures were then incubated at 22°C and 28°C under continuous shaking conditions. After enrichment, 100 µL of the cultures were spread on the R2A/BMB agar plate. The 16S rDNA gene of the colonies was amplified using standard 16S primers. The amplified fragments were Sanger sequenced afterward at Eurofins (Elsberg, Germany). The colonies were then screened for their hydrolysis activity on tributyrin (TBT), polycaprolactone (PCL), and bis-(2-hydroxyethyl) terephthalate (BHET). The strains with hydrolytic activities observed toward TBT, PCL, or BHET were later selected for whole-genome sequencing. Genomic DNA (gDNA) was extracted with the NucleoSpin Microbial DNA Kit from MN (Düren, Germany) from 5 mL cultures and then sequenced using Illumina NextSeq 500 sequencing method at Eurofins (Germany).

Bacterial strains and plasmids used in the study are listed in Table S5. *E. coli* was grown in LB medium (1% tryptone/peptone, 0.5% yeast extract, and 1% NaCl) supplemented with appropriate antibiotics at 250 rpm in flasks under aerobic conditions at 37°C for 20 h.

### Data availability and bioinformatic analysis

The genome of isolates UHH-5R5 and UHH-Hm9b that originated from marine aquaculture was submitted to IMG.gov under the submission IDs 294449 and 294450, respectively. The sequence reads were assembled using SPAdes (v.3.15.0) (48) and initially annotated with Prokka (v.1.14.6) (49). To identify putative PET esterases within the *Bacteroidetes* genome data sets, a profile hidden Markov model (HMM) was constructed based on the known, functionally tested enzymes (50). The HMM analysis identified PET93 and PET94 as homologs of known PET-degrading enzymes, which were subsequently investigated further.

Nucleotide and amino acid sequences of putative PET esterases were obtained from genomic data of isolates UHH-5R5 and UHH-Hm9b from IMG. Sequence data were processed and analyzed using Snapgene (GSL Biotech LLC, San Diego, CA, USA). Conserved domains in the protein sequences were identified using CD-search (51). A phylogenetic tree was constructed with MEGA-X, employing maximum bootstrap of 1,000 for enhanced accuracy (52). Structural information was retrieved from the RCSB-PDB database (53), and protein structures were predicted using AlphaFold2 with default parameters (54). The 3D protein models were visualized in UCSF Chimera; further, the structural alignment with homologous proteins was generated with Chimera MatchMaker tool (55). SignalP 5.0 server was used to predict the native signal peptide sequences (56).

### Biofilms on PET surface: growth and degradation product analysis

Precultures of UHH-5R5 and UHH-Hm9b were inoculated in BMB media at 28°C with continuous shaking at 130 rpm. The cultures were then diluted to an OD_600_ of 0.05, and then 5 mL of each diluted culture was transferred to a six-well plate (Nunc cell culture plate, catalog no. 130184; Thermo Fisher Scientific, Waltham, MA, USA). PET foil platelets (Ø 35 mm, amorphous PET film, Goodfellow GmbH, Bad Nauheim, Germany) were sterilized in Ethanol 70% for 10 min and then added to each well, which were subsequently incubated at 28°C under 80 rpm shaking condition to facilitate biofilm formation. Supernatant was collected after 3, 5, and 7 days of incubation. Each 10 mL of supernatant in each sample was vacuum dried to a final volume of 600 µL.

The concentrated supernatants were further analyzed using UltiMate 3000 UHPLC system (Thermo Fisher Scientific). The Triart C18 column (YMC Europe GmbH, Dinslaken, Germany), 100 × 2.0 mm with 1.9 µm diameter was employed for separation. Isocratic elution was performed with a mobile phase consisting of 20:80 (vol/vol) acetonitrile and water (acidified with 0.1% vol/vol trifluoroacetic acid) at a flow rate of 0.4 mL min^−1^. Fifty microliters of concentrated supernatant was mixed with 200 µL of acetonitrile (acidified with 1% vol trifluoroacetic acid), followed by centrifugation at 10,000 × *g* for 3 min. A 200 µL aliquot of the mixture was then diluted with 600 µL water. Each 15 µL of sample was then injected for each measurement. As negative controls, PET foils are incubated in media without bacterial cells, and only medium was incubated under the same conditions as *E. coli* Dh5α. These samples were taken at the same time point. All experiments were performed with three biological replicates.

### Imaging analysis of biofilms on PET foil platelets

For observation of biofilm formation of Bacteroidetes isolates UHH-5R5 and UHH-Hm9b on plastic surface, the cells were grown in BMB medium at 28°C with 130 rpm shaking until reaching an optical cell density (OD_600_) of 1. These starter cultures were then diluted to OD_600_ of 0.05 in fresh BMB medium. The cultures were incubated with PET foils at 28°C with 80 rpm shaking. After incubation, foil was washed three times with 1× PBS buffer and placed to μ-Slide eight-well plates (ibiTreat, catalog no. 80826, ibidi USA, Inc., Fitchburg, WI, USA). The cells were stained using 100 µL of the LIVE/DEAD BacLight Bacterial Viability Kit (Thermo Scientific). The cells were then analyzed using the Axio Observer Z1/7, LSM 800 confocal microscope equipped with an objective C-Apochromat 63×/1.2 W Korr UV VisIR (Carl Zeiss Microscopy GmbH, Jena, Germany), utilizing the SYTO-9 channel (emission wavelength: 528/20 nm) and the PI channel (emission wavelength: 645/20 nm). For the analysis of the CLSM images, the ZEN software was used (version 2.3, Carl Zeiss Microscopy GmbH). For each sample, at least three different positions were observed, and one representative CLSM image was chosen.

### Heterologous expression of recombinant putative enzymes

PET93 and PET94 were amplified from the genomic DNA of UHH-5R5 and UHH-Hm9b and cloned into the pET21a (+) vector. The constructs were sequenced at Microsynth Seqlab GmbH (Göttingen, Germany) and compared to the original sequence to check for the correctness. Sequences coding mature PET94 (sequence devoid of the signal peptide) and PET93 protein were heterologously expressed in *E. coli* BL21 (DE3) using *β*-d-1-thiogalactopyranoside (IPTG) induction. Cultures were incubated aerobically in LB medium with ampicillin 100 μg/mL at 37°C. When OD_600_ reached 0.7–0.8, the expressions were induced with IPTG 1 mM, followed by incubation at 22°C and 17°C for 20 h. The cells were harvested and lysed with pressure using a French press. Afterward, the proteins with C-terminal 6x histidine tag were purified via Nickel-ion affinity chromatography using Ni-NTA agarose (Qiagen, Hilden, Germany) and analyzed by SDS-PAGE. The elution buffer was exchanged against 0.1 mM potassium phosphate buffer pH 7.0 in a 30 kDa Amicon Tube (GE HealthCare, Solingen, Germany).

### Plate-based activity assay and partial biochemical characterization of PET93 and PET94

For activity tests, purified recombinant proteins were utilized. Agar plates were prepared containing 10 mM bis-(2-hydroxyethyl) terephthalate (BHET) and 500 mg L^−1^ polycaprolactone (PCL). Ten microliters of eluate from protein purification was spotted onto the plates to observe the halo formation.

For the *p*NP assay, unless otherwise specified, 0.1–1 µg of the enzyme was added to a substrate solution containing 190 µL of 0.1 M potassium phosphate (pH 7–8) and 10 µL of 0.1 mM pNP-substrates dissolved in isopropanol. The reaction was terminated after 10 min by adding 200 mM of Na_2_CO_3_. The samples were then centrifuged at 4°C and 13,000 rpm for 3 min. Various *p*NP ester substrates with chain lengths of C4, C6, C8, C10, C12, C14, C16, and C18 were tested. Enzyme activity was indicated by the color change from colorless to yellow, with the absorbance measured at 405 nm in a plate reader (BioTek, Winooski, VT, USA). All measurements were performed in triplicate. The optimal temperature for enzyme activity was evaluated between 10°C and 90°C. Additionally, the effect of pH on enzyme activity was assessed using citrate phosphate buffer (pH 3.0, 4.0, and 5.0), potassium phosphate buffer (pH 6.0, 7.0, and 8.0), and carbonate bicarbonate buffer (pH 9.2 and 10.2) with pNP-C8 as the substrate.

### UHPLC-based activity assay for PET, MHET, and BHET degradation

Purified proteins at the concentration of 0.1 mg mL^−1^ were incubated with MHET (1 mM), BHET (5 mM), PET powder (10 g mL^−1^), PET foil/powder, and UV-treated PET foil/powder in 200 µL of potassium phosphate buffer (pH 7–8). After 24 h of incubation at 37°C with MHET/BHET and 120 h with PET, the supernatant was filtered through 0.22 µm filter paper. The release of TPA was then analyzed using UHPLC and TPA reporter strain *C. thiooxidans* UHH04 (28). BSA incubated with PET substrates under the same conditions served as a negative control.

### *C. thiooxidans* S23 reporter strain preparation for TPA assays

The protocol for this experiment was adapted from reference (28). The *C. thiooxidans* S23 biosensor strain ReporTPA_UHH04 was incubated overnight at 130 rpm in 50 mL LB medium containing 25 µg/mL chloramphenicol and additionally supplemented with 10 mM gluconate in an Erlenmeyer flask. Before performing the TPA assays, the OD_600_ of the cultures was measured, and an appropriate volume was centrifuged at 4,500 rcf and 4°C for 5 min. The resulting pellet was resuspended in 50 mL Wx medium containing 25 µg/mL chloramphenicol to achieve a final OD_600_ of 0.6. The resuspended cultures were incubated at 37°C and 130 rpm for 30 min before being added to the samples.

For standard assay, 100 µL of the sample was added to each well of a black-walled 96-well microtiter plate (ThermoFisher, Waltham, MA, USA) designed for fluorescence-based assays. An additional 100 µL of reporter cells UHH04, prepared as described above, was added to each sample well. The plate was incubated at 28°C on a Vibration Shaker 3023 (Gesellschaft für Labortechnik mbH, Burgwedel, Germany) at 150 rpm. Fluorescence and OD_600_ measurements were taken at intervals of 0.5–2 h using a Synergy HT plate reader with Gen5 software (BioTek).

### Fluorescence microscopy of *C. thiooxidans* S23 reporter strain

Microscopic imaging of reporter cells was performed with a confocal laser scanning microscope (Axio Observer.Z1/7 LSM 800; Carl Zeiss Microscopy GmbH) using Plan-Apochromat 100×/1.40 Oil DIC M27 objective. Image analysis and processing were carried out using ZEN software (Version 2.3, Carl Zeiss Microscopy GmbH).

### Global distribution of PET93 and PET94 homologs

The IMG/M scans for PET93 and PET94 homologs were completed on 20 April 2025. When available, Geo locations were used as provided on IMG. In case the data were missing, we attempted to retrieve Geo coordinates using details about isolation source/location/city/country on the IMG database. The map illustrates both the frequency and geographical distribution of the homologs of dienelactone hydrolases in the strains UHH-5R5 and UHH-Hm9b and was created using the Cartopy Python package (version 0.24.0), which is freely available at https://scitools.org.uk/cartopy. A similarity threshold of 50% was applied in homology searches. Only bacterial hits classified within the Bacteroidota phylum were included in the final data set.

### Proteome identification of PET93 and PET94 in UHH-5R5 and UHH-Hm9b

For the proteome analysis, strains UHH-5R5 and UHH-Hm9b were grown overnight in artificial seawater supplemented with 25 mL L^−1^ yeast-extract-peptone (YP). These cultures were used to inoculate biofilm and planktonic conditions.

For biofilm cultivation, ethanol-pre-sterilized, PET foils were cut to fit the bottom of six-well plates. Separate plates were prepared with and without PET foil. Each condition was set up in biological duplicates. Three wells per condition were filled with 4 mL medium and inoculated to an initial OD_600_ of 0.05. Plates were incubated for 5 days at 28°C with shaking at 60 rpm.

For planktonic cultivation, 20 mL medium was dispensed into Erlenmeyer flasks and inoculated to an initial OD_600_ of 0.05. Cultures were grown with or without 1 mM bis(2-hydroxyethyl) terephthalate (BHET). BHET was added from a DMSO stock solution. Cultures were incubated for 5 days at 28°C with shaking at 130 rpm.

After incubation, biofilm cells were detached from the PET surface using a sterile cell scraper. Biomass from three wells (4 mL each) was pooled into a 50 mL centrifuge tube. Samples were centrifuged at 5,000 rpm for 15 min at 4°C. The supernatant was discarded. Pellets were washed three times with 1× phosphate-buffered saline (PBS). Planktonic cells were harvested and processed identically, except that no scraping step was required. After the final wash, cell pellets were collected and used for proteomic analysis.

## SUPPLEMENTAL MATERIAL

Supplemental tables aem.01698-25-s0001.docx Tables S1 to S3, S5, and S6.

Table S4 aem.01698-25-s0002.xlsx Proteome data.

ASM does not own the copyrights to Supplemental Material that may be linked to, or accessed through, an article. The authors have granted ASM a non-exclusive, world-wide license to publish the Supplemental Material files. Please contact the corresponding author directly for reuse.

## References

[1] Eriksen M, Lebreton LCM, Carson HS, Thiel M, Moore CJ, Borerro JC, Galgani F, Ryan PG, Reisser J. 2014. Plastic pollution in the world's oceans: more than 5 trillion plastic pieces weighing over 250,000 tons afloat at sea. PLoS One 9:e111913. doi:10.1371/journal.pone.0111913 25494041 PMC4262196

[2] Jambeck JR, Geyer R, Wilcox C, Siegler TR, Perryman M, Andrady A, Narayan R, Law KL. 2015. Marine pollution. Plastic waste inputs from land into the ocean. Science 347:768–771. doi:10.1126/science.1260352 25678662

[3] Nayanathara Thathsarani Pilapitiya PGC, Ratnayake AS. 2024. The world of plastic waste: a review. Cleaner Materials 11:100220. doi:10.1016/j.clema.2024.100220

[4] OECD. 2022. Global plastics outlook: policy scenarios to 2060. OECD Publishing, Paris.

[5] Chow J, Perez-Garcia P, Dierkes R, Streit WR. 2023. Microbial enzymes will offer limited solutions to the global plastic pollution crisis. Microb Biotechnol 16:195–217. doi:10.1111/1751-7915.14135 36099200 PMC9871534

[6] Kaandorp MLA, Lobelle D, Kehl C, Dijkstra HA, van Sebille E. 2023. Global mass of buoyant marine plastics dominated by large long-lived debris. Nat Geosci 16:689–694. doi:10.1038/s41561-023-01216-0

[7] Osman AI, Hosny M, Eltaweil AS, Omar S, Elgarahy AM, Farghali M, Yap PS, Wu YS, Nagandran S, Batumalaie K, Gopinath SCB, John OD, Sekar M, Saikia T, Karunanithi P, Hatta MHM, Akinyede KA. 2023. Microplastic sources, formation, toxicity and remediation: a review. Environ Chem Lett:1–41. doi:10.1007/s10311-023-01593-3 PMC10072287 37362012

[8] Wright RJ, Bosch R, Gibson MI, Christie-Oleza JA. 2020. Plasticizer degradation by marine bacterial isolates: a proteogenomic and metabolomic characterization. Environ Sci Technol 54:2244–2256. doi:10.1021/acs.est.9b05228 31894974 PMC7031849

[9] Pérez-García P, Sass K, Wongwattanarat S, Amann J, Feuerriegel G, Neumann T, Bäse N, Schmitz LS, Dierkes RF, Gurschke MF, Wypych A, Bounabi H, de Divitiis M, Vollstedt C, Streit WR. 2025. Microbial plastic degradation: enzymes, pathways, challenges, and perspectives. Microbiol Mol Biol Rev 89:e0008724. doi:10.1128/mmbr.00087-24 40970732 PMC12713388

[10] Buchholz PCF, Feuerriegel G, Zhang H, Perez-Garcia P, Nover LL, Chow J, Streit WR, Pleiss J. 2022. Plastics degradation by hydrolytic enzymes: the plastics-active enzymes database-PAZy. Proteins 90:1443–1456. doi:10.1002/prot.26325 35175626

[11] Danso D, Chow J, Streit WR. 2019. Plastics: environmental and biotechnological perspectives on microbial degradation. Appl Environ Microbiol 85:e01095-19. doi:10.1128/AEM.01095-19 31324632 PMC6752018

[12] Tournier V, Duquesne S, Guillamot F, Cramail H, Taton D, Marty A, André I. 2023. Enzymes' power for plastics degradation. Chem Rev 123:5612–5701. doi:10.1021/acs.chemrev.2c00644 36916764

[13] Wei R, Zimmermann W. 2017. Microbial enzymes for the recycling of recalcitrant petroleum-based plastics: how far are we? Microb Biotechnol 10:1308–1322. doi:10.1111/1751-7915.12710 28371373 PMC5658625

[14] Wright RJ, Langille MGI, Walker TR. 2021. Food or just a free ride? A meta-analysis reveals the global diversity of the Plastisphere. ISME J 15:789–806. doi:10.1038/s41396-020-00814-9 33139870 PMC8027867

[15] Lapébie P, Lombard V, Drula E, Terrapon N, Henrissat B. 2019. Bacteroidetes use thousands of enzyme combinations to break down glycans. Nat Commun 10:2043. doi:10.1038/s41467-019-10068-5 31053724 PMC6499787

[16] Krieg NR, Ludwig W, Euzéby JP, Whitman WB. 2015. Bacteroidetes phyl, p 1–2. In Bergey’s manual of systematics of Archaea and Bacteria

[17] Mann AJ, Hahnke RL, Huang S, Werner J, Xing P, Barbeyron T, Huettel B, Stüber K, Reinhardt R, Harder J, Glöckner FO, Amann RI, Teeling H. 2013. The genome of the alga-associated marine flavobacterium Formosa agariphila KMM 3901T reveals a broad potential for degradation of algal polysaccharides. Appl Environ Microbiol 79:6813–6822. doi:10.1128/AEM.01937-13 23995932 PMC3811500

[18] Yoon B-J, Oh D-C. 2012. Spongiibacterium flavum gen. nov., sp. nov., a member of the family Flavobacteriaceae isolated from the marine sponge Halichondria oshoro, and emended descriptions of the genera Croceitalea and Flagellimonas. Int J Syst Evol Microbiol 62:1158–1164. doi:10.1099/ijs.0.027243-0 21724954

[19] Dudek KL, Cruz BN, Polidoro B, Neuer S. 2020. Microbial colonization of microplastics in the Caribbean Sea. Limnol Oceanogr Letters 5:5–17. doi:10.1002/lol2.10141

[20] Pinto M, Polania Zenner P, Langer TM, Harrison J, Simon M, Varela MM, Herndl GJ. 2020. Putative degraders of low-density polyethylene-derived compounds are ubiquitous members of plastic-associated bacterial communities in the marine environment. Environ Microbiol 22:4779–4793. doi:10.1111/1462-2920.15232 32935476 PMC7702132

[21] Vaksmaa A, Knittel K, Abdala Asbun A, Goudriaan M, Ellrott A, Witte HJ, Vollmer I, Meirer F, Lott C, Weber M, Engelmann JC, Niemann H. 2021. Microbial communities on plastic polymers in the Mediterranean sea. Front Microbiol 12:673553. doi:10.3389/fmicb.2021.673553 34220756 PMC8243005

[22] Danso D, Schmeisser C, Chow J, Zimmermann W, Wei R, Leggewie C, Li X, Hazen T, Streit WR. 2018. New insights into the function and global distribution of polyethylene terephthalate (PET)-degrading bacteria and enzymes in marine and terrestrial metagenomes. Appl Environ Microbiol 84:e02773-17. doi:10.1128/AEM.02773-17 29427431 PMC5881046

[23] Dierkes RF, Wypych A, Pérez-García P, Danso D, Chow J, Streit WR. 2023. An ultra-sensitive Comamonas thiooxidans biosensor for the rapid detection of enzymatic polyethylene terephthalate (PET) degradation. Appl Environ Microbiol 89:e0160322. doi:10.1128/aem.01603-22 36507653 PMC9888244

[24] Zhang H, Perez-Garcia P, Dierkes RF, Applegate V, Schumacher J, Chibani CM, Sternagel S, Preuss L, Weigert S, Schmeisser C, Danso D, Pleiss J, Almeida A, Höcker B, Hallam SJ, Schmitz RA, Smits SHJ, Chow J, Streit WR. 2022. The bacteroidetes Aequorivita sp. and Kaistella jeonii produce promiscuous esterases with PET-hydrolyzing activity. Front Microbiol 12. doi:10.3389/fmicb.2021.803896 PMC8767016 35069509

[25] Chen I-M, Chu K, Palaniappan K, Ratner A, Huang J, Huntemann M, Hajek P, Ritter SJ, Webb C, Wu D, Varghese NJ, Reddy TBK, Mukherjee S, Ovchinnikova G, Nolan M, Seshadri R, Roux S, Visel A, Woyke T, Eloe-Fadrosh EA, Kyrpides NC, Ivanova NN. 2023. The IMG/M data management and analysis system v.7: content updates and new features. Nucleic Acids Res 51:D723–D732. doi:10.1093/nar/gkac976 36382399 PMC9825475

[26] Mukherjee S, Stamatis D, Li CT, Ovchinnikova G, Bertsch J, Sundaramurthi JC, Kandimalla M, Nicolopoulos PA, Favognano A, Chen I-M, Kyrpides NC, Reddy TBK. 2023. Twenty-five years of Genomes OnLine Database (GOLD): data updates and new features in v.9. Nucleic Acids Res 51:D957–D963. doi:10.1093/nar/gkac974 36318257 PMC9825498

[27] Ge H, Li C, Huang C, Zhao L, Cong B, Liu S. 2025. Bacterial community composition and metabolic characteristics of three representative marine areas in northern China. Mar Environ Res 204:106892. doi:10.1016/j.marenvres.2024.106892 39647426

[28] Alejandre-Colomo C, Francis B, Viver T, Harder J, Fuchs BM, Rossello-Mora R, Amann R. 2021. Cultivable Winogradskyella species are genomically distinct from the sympatric abundant candidate species. ISME Commun 1:51. doi:10.1038/s43705-021-00052-w 36747039 PMC9723794

[29] Avcı B, Krüger K, Fuchs BM, Teeling H, Amann RI. 2020. Polysaccharide niche partitioning of distinct Polaribacter clades during North Sea spring algal blooms. ISME J 14:1369–1383. doi:10.1038/s41396-020-0601-y 32071394 PMC7242417

[30] Khan SA, Jeong SE, Baek JH, Jeon CO. 2020. Maribacter algicola sp. nov., isolated from a marine red alga, Porphyridium marinum, and transfer of Maripseudobacter aurantiacus Chen et al. 2017 to the genus Maribacter as Maribacter aurantiacus comb. nov. Int J Syst Evol Microbiol 70:797–804. doi:10.1099/ijsem.0.003828 31682218

[31] López-Sánchez R, Rebollar EA, Gutiérrez-Ríos RM, Garciarrubio A, Juarez K, Segovia L. 2024. Metagenomic analysis of carbohydrate-active enzymes and their contribution to marine sediment biodiversity. World J Microbiol Biotechnol 40:95. doi:10.1007/s11274-024-03884-5 38349445 PMC10864421

[32] Lu D-C, Wang F-Q, Amann RI, Teeling H, Du Z-J. 2023. Epiphytic common core bacteria in the microbiomes of co-located green (Ulva), brown (Saccharina) and red (Grateloupia, Gelidium) macroalgae. Microbiome 11:126. doi:10.1186/s40168-023-01559-1 37264413 PMC10233909

[33] Sun C, Zhao W, Yue W, Cheng H, Long A, Yin J, Sun F, Wang Y. 2026. Degradation of polymeric carbohydrates coupled with cellular motility driving microbial niche separation in the Pearl River Estuary surface sediment. J Environ Sci (China) 160:414–423. doi:10.1016/j.jes.2025.04.032 41177622

[34] Bakunina I, Nedashkovskaya O, Balabanova L, Zvyagintseva T, Rasskasov V, Mikhailov V. 2013. Comparative analysis of glycoside hydrolases activities from phylogenetically diverse marine bacteria of the genus Arenibacter. Mar Drugs 11:1977–1998. doi:10.3390/md11061977 23752354 PMC3721217

[35] Gao J-W, Ying J-J, Dong H, Liu W-J, He D-Y, Xu L, Sun C. 2023. Characterization of Maribacter polysaccharolyticus sp. nov., Maribacter huludaoensis sp. nov., and Maribacter zhoushanensis sp. nov. and illumination of the distinct adaptative strategies of the genus Maribacter. Front Mar Sci 10:2023. doi:10.3389/fmars.2023.1248754

[36] Kalenborn S, Zühlke D, Reintjes G, Riedel K, Amann RI, Harder J. 2024. Genes for laminarin degradation are dispersed in the genomes of particle-associated Maribacter species. Front Microbiol 15:1393588. doi:10.3389/fmicb.2024.1393588 39188312 PMC11345257

[37] Wolter LA, Mitulla M, Kalem J, Daniel R, Simon M, Wietz M. 2021. CAZymes in Maribacter dokdonensis 62–1 from the patagonian shelf: genomics and physiology compared to related flavobacteria and a co-occurring Alteromonas strain. Front Microbiol 12:628055. doi:10.3389/fmicb.2021.628055 33912144 PMC8072126

[38] Du Y, Liu X, Dong X, Yin Z. 2022. A review on marine plastisphere: biodiversity, formation, and role in degradation. Comput Struct Biotechnol J 20:975–988. doi:10.1016/j.csbj.2022.02.008 35242288 PMC8861569

[39] Marques J, Ares A, Costa J, Marques MPM, de Carvalho L, Bessa F. 2023. Plastisphere assemblages differ from the surrounding bacterial communities in transitional coastal environments. Sci Total Environ 869:161703. doi:10.1016/j.scitotenv.2023.161703 36708826

[40] Amaral-Zettler LA, Zettler ER, Mincer TJ. 2020. Ecology of the plastisphere. Nat Rev Microbiol 18:139–151. doi:10.1038/s41579-019-0308-0 31937947

[41] Qian Y, Huang L, Yan P, Wang X, Luo Y. 2024. Biofilms on plastic debris and the microbiome. Microorganisms 12:1362. doi:10.3390/microorganisms12071362 39065130 PMC11278848

[42] Vaksmaa A, Hernando-Morales V, Zeghal E, Niemann H. 2021. Microbial degradation of marine plastics: current state and future prospects, p 111–154. In Joshi SJ, Deshmukh A, Sarma H (ed), Biotechnology for sustainable environment. Springer Singapore, Singapore.

[43] Yang Y, Liu W, Zhang Z, Grossart H-P, Gadd GM. 2020. Microplastics provide new microbial niches in aquatic environments. Appl Microbiol Biotechnol 104:6501–6511. doi:10.1007/s00253-020-10704-x 32500269 PMC7347703

[44] Dutschei T, Beidler I, Bartosik D, Seeßelberg J-M, Teune M, Bäumgen M, Ferreira SQ, Heldmann J, Nagel F, Krull J, Berndt L, Methling K, Hein M, Becher D, Langer P, Delcea M, Lalk M, Lammers M, Höhne M, Hehemann J-H, Schweder T, Bornscheuer UT. 2023. Marine Bacteroidetes enzymatically digest xylans from terrestrial plants. Environ Microbiol 25:1713–1727. doi:10.1111/1462-2920.16390 37121608

[45] Kabisch A, Otto A, König S, Becher D, Albrecht D, Schüler M, Teeling H, Amann RI, Schweder T. 2014. Functional characterization of polysaccharide utilization loci in the marine Bacteroidetes 'Gramella forsetii' KT0803. ISME J 8:1492–1502. doi:10.1038/ismej.2014.4 24522261 PMC4069401

[46] Tang K, Lin Y, Han Y, Jiao N. 2017. Characterization of potential polysaccharide utilization systems in the marine Bacteroidetes Gramella flava JLT2011 using a multi-omics approach. Front Microbiol 8:220. doi:10.3389/fmicb.2017.00220 28261179 PMC5306329

[47] Preuss L, Alawi M, Dumnitch A, Trinh L, Maison W, Burmeister N, Poehlein A, Daniel R, Vollstedt C, Streit WR. 2025. Polyethylene terephthalate (PET) primary degradation products affect c-di-GMP-, cAMP-signaling, and quorum sensing (QS) in Vibrio gazogenes DSM 21264. Microbiol Spectr 13:e0018125. doi:10.1128/spectrum.00181-25 40488468 PMC12211005

[48] Bankevich A, Nurk S, Antipov D, Gurevich AA, Dvorkin M, Kulikov AS, Lesin VM, Nikolenko SI, Pham S, Prjibelski AD, Pyshkin AV, Sirotkin AV, Vyahhi N, Tesler G, Alekseyev MA, Pevzner PA. 2012. SPAdes: a new genome assembly algorithm and its applications to single-cell sequencing. J Comput Biol 19:455–477. doi:10.1089/cmb.2012.0021 22506599 PMC3342519

[49] Seemann T. 2014. Prokka: rapid prokaryotic genome annotation. Bioinformatics 30:2068–2069. doi:10.1093/bioinformatics/btu153 24642063

[50] Mistry J, Finn RD, Eddy SR, Bateman A, Punta M. 2013. Challenges in homology search: HMMER3 and convergent evolution of coiled-coil regions. Nucleic Acids Res 41:e121. doi:10.1093/nar/gkt263 23598997 PMC3695513

[51] Marchler-Bauer A, Bryant SH. 2004. CD-Search: protein domain annotations on the fly. Nucleic Acids Res 32:W327–W331. doi:10.1093/nar/gkh454 15215404 PMC441592

[52] Kumar S, Stecher G, Li M, Knyaz C, Tamura K. 2018. MEGA X: molecular evolutionary genetics analysis across computing platforms. Mol Biol Evol 35:1547–1549. doi:10.1093/molbev/msy096 29722887 PMC5967553

[53] Berman HM, Westbrook J, Feng Z, Gilliland G, Bhat TN, Weissig H, Shindyalov IN, Bourne PE. 2000. The Protein Data Bank. Nucleic Acids Res 28:235–242. doi:10.1093/nar/28.1.235 10592235 PMC102472

[54] Jumper J, Evans R, Pritzel A, Green T, Figurnov M, Ronneberger O, Tunyasuvunakool K, Bates R, Žídek A, Potapenko A, et al.. 2021. Highly accurate protein structure prediction with AlphaFold. Nature 596:583–589. doi:10.1038/s41586-021-03819-2 34265844 PMC8371605

[55] Pettersen EF, Goddard TD, Huang CC, Meng EC, Couch GS, Croll TI, Morris JH, Ferrin TE. 2021. UCSF ChimeraX: structure visualization for researchers, educators, and developers. Protein Sci 30:70–82. doi:10.1002/pro.3943 32881101 PMC7737788

[56] Almagro Armenteros JJ, Tsirigos KD, Sønderby CK, Petersen TN, Winther O, Brunak S, von Heijne G, Nielsen H. 2019. SignalP 5.0 improves signal peptide predictions using deep neural networks. Nat Biotechnol 37:420–423. doi:10.1038/s41587-019-0036-z 30778233

