# Supplementary material for: Role of dienelactone hydrolases in PET biodegradation by flavobacteria Maribacter dokdonensis and Arenibacter palladensis
Source: Appl Environ Microbiol. 2026 Apr 24;92(5):e01698-25. doi: 10.1128/aem.01698-25 (PMC13188925; doi:10.1128/aem.01698-25)
Supplement: Supplemental tables — Tables S1 to S3, S5, and S6. [file aem.01698-25-s0001.docx]

***Supplementary Tables S1-S4 and S5&6:**

**Table S1:** Quantitative analysis of biofilm morphology formed by the Bacteroidota isolates UHH-5R5 and UHH-Hm9b on PET foil over a 7-day incubation period using BiofilmQ. Representative data of *n = 3* independent biofilms for each treatment are presented.

**Table S2:** Predicted lipases and esterases identified in UHH-5R5 genome and their secretion-related features

**Table S3:** Predicted lipases and esterases identified in UHH-Hm9b genome and their secretion-related feature

**Table S4:** Proteome data set of ofUHH-5R5 and UHH-Hm9b grown under planktonic and biofilm conditions (Excel-File).

**Table S5:** Bacterial strains and plasmids used in this study

**Table S6:** Homologs of PET93 and PET94 hydrolases from Bacteroidota isolates UHH-5R5 and UHH-Hm9b. Data were retrieved from the publicly available IMG/MER database, applying thresholds of ≥50% sequence identity and ≥80% sequence coverage.

**Tables S1-3,5,6 are supplied as Word file and Table S4 as an Excel file*.

**Table S1:** Quantitative analysis of biofilm morphology formed by the Bacteroidota isolates UHH-5R5 and UHH-Hm9b on PET foil over a 7-day incubation period using BiofilmQ. Representative data of *n = 3* independent biofilms for each treatment are presented.

| File name | Biofilm HeighttoLength (a.u.) | Biofilm HeightToWidth (a.u.) | Biofilm LengthToWidth (a.u.) | Biofilm_Height  (µm) | Biofilm_Length  (µm) | Biofilm_MeanThickness  (µm) | Biofilm_Volume  (µm³) | Biofilm_Width  (µm) |
| --- | --- | --- | --- | --- | --- | --- | --- | --- |
| UHH-Hm9b | | | | | | | | |
| Day 2_1 | 0.149 | 0.148 | 0.995 | 17.970 | 120.492 | 9.641 | 516.638 | 121.111 |
| Day 2_2 | 0.138 | 0.123 | 0.886 | 17.445 | 126.046 | 7.846 | 2180.643 | 142.210 |
| Day 2_3 | 0.159 | 0.126 | 0.797 | 17.588 | 110.796 | 7.811 | 895.251 | 139.082 |
| Day 3_1 | 0.179 | 0.192 | 1.072 | 23.766 | 132.779 | 14.158 | 3952.286 | 123.923 |
| Day 3_2 | 0.207 | 0.220 | 1.059 | 26.950 | 129.891 | 8.250 | 686.550 | 122.704 |
| Day 3_3 | 0.185 | 0.234 | 1.265 | 27.631 | 149.093 | 13.594 | 7164.869 | 117.871 |
| Day 6_1 | 0.227 | 0.201 | 0.884 | 27.500 | 120.993 | 13.847 | 4001.465 | 136.920 |
| Day 6_2 | 0.138 | 0.139 | 1.010 | 17.562 | 127.373 | 11.973 | 1690.761 | 126.103 |
| Day 6_3 | 0.220 | 0.162 | 0.740 | 25.210 | 114.850 | 14.458 | 3133.971 | 155.285 |
| Day 7_1 | 0.208 | 0.225 | 1.083 | 27.701 | 133.384 | 13.814 | 2955.924 | 123.141 |
| Day 7_2 | 0.247 | 0.222 | 0.901 | 27.756 | 112.514 | 12.820 | 168.790 | 124.934 |
| Day 7_3 | 0.236 | 0.224 | 0.950 | 27.649 | 117.053 | 18.754 | 989.514 | 123.256 |
| UHH-5R5 | | | | | | | | |
| Day 2_1 | 0.095 | 0.078 | 0.827 | 10.218 | 107.691 | 6.822 | 2308.372 | 130.280 |
| Day 2_2 | 0.045 | 0.031 | 0.682 | 4.830 | 107.507 | 4.638 | 535.207 | 157.634 |
| Day 2_3 | 0.057 | 0.079 | 1.389 | 7.876 | 139.261 | 6.318 | 693.113 | 100.298 |
| Day 3_1 | 0.103 | 0.093 | 0.902 | 12.255 | 118.500 | 5.743 | 4800.371 | 131.320 |
| Day 3_2 | 0.106 | 0.086 | 0.814 | 12.490 | 118.239 | 5.362 | 4166.769 | 145.258 |
| Day 3_3 | 0.164 | 0.146 | 0.892 | 19.327 | 118.140 | 9.191 | 915.154 | 132.372 |
| Day 6_1 | 0.218 | 0.210 | 0.960 | 27.540 | 126.147 | 16.111 | 8821.380 | 131.399 |
| Day 6_2 | 0.183 | 0.225 | 1.225 | 27.256 | 148.627 | 15.079 | 10738.695 | 121.354 |
| Day 6_3 | 0.182 | 0.214 | 1.176 | 25.552 | 140.453 | 12.470 | 643.019 | 119.436 |
| Day 7_1 | 0.148 | 0.252 | 1.708 | 27.694 | 187.544 | 10.714 | 5502.643 | 109.809 |
| Day 7_2 | 0.171 | 0.209 | 1.223 | 23.544 | 137.758 | 8.479 | 2288.972 | 112.649 |
| Day 7_3 | 0.199 | 0.223 | 1.123 | 27.118 | 136.300 | 14.975 | 10349.788 | 121.350 |

**Table S2:** Predicted lipases and esterases identified in UHH-5R5 genome

| Locus tag | Gene product name | Signal peptide | Transmembrane Helices |
| --- | --- | --- | --- |
| Ga0596861_0001_587820_588374 | Serine hydrolase | No | No |
| Ga0596861_0001_674084_675001 | Predicted alpha/beta hydrolase | No | No |
| Ga0596861_0001_1092288_1093181 | Epoxide hydrolase 4 | No | No |
| Ga0596861_0003_249026_250111 | Epsilon-lactone hydrolase | No | No |
| Ga0596861_0003_282091_283320 | Uncharacterized OsmC-like protein / alpha/beta superfamily hydrolase | No | No |
| Ga0596861_0003_429351_430088 | Dienelactone hydrolase | Yes | No |
| Ga0596861_0003_663162_664571 | Predicted dienelactone hydrolase | Yes | Yes |
| Ga0596861_0004_94874_96127 | Predicted alpha/beta superfamily hydrolase | No | Yes |
| Ga0596861_0005_483643_484587 | Alpha-beta hydrolase superfamily lysophospholipase | No | No |
| Ga0596861_0006_278382_279587 | Uncharacterized OsmC-like protein / alpha/beta superfamily hydrolase | No | No |
| Ga0596861_0291_2_193 | Hydroxyacylglutathione hydrolase-like protein | No | No |
| Ga0596861_0467_1_384 | Alpha/beta superfamily hydrolase | No | No |
| Ga0596861_0933_1_300 | Alkyl sulfatase BDS1-like metallo-beta-lactamase superfamily hydrolase | No | No |
| Ga0596861_0977_1_291 | Alpha-beta hydrolase superfamily lysophospholipase | No | No |
| Ga0596861_1363_3_266 | Alpha-beta hydrolase superfamily lysophospholipase | No | No |
| Ga0596861_1732_1_252 | Dienelactone hydrolase | No | No |
| Ga0596861_1587_3_257 | Triacylglycerol esterase/lipase EstA (α/β hydrolase) | No | No |
| Ga0596861_0018_47238_47897 | Phospholipase/carboxylesterase | No | No |
| Ga0596861_0006_60018_62606 | Lysophospholipase L1-like esterase | No | No |
| Ga0596861_0017_36371_37060 | Lysophospholipase L1-like esterase | No | No |
| Ga0596861_0011_84374_85063 | Lysophospholipase L1-like esterase | Yes | No |
| Ga0596861_0010_116659_117408 | Lysophospholipase L1-like esterase | No | Yes |
| Ga0596861_0005_83462_84232 | Lysophospholipase L1-like esterase | No | Yes |
| Ga0596861_0001_151967_152605 | Lysophospholipase L1-like esterase | No | No |
| Ga0596861_0004_623245_623970 | Lysophospholipase L1-like esterase | Yes | No |
| Ga0596861_1654_3_254 | Predicted acylesterase/phospholipase RssA | No | No |
| Ga0596861_0006_426390_427166 | Esterase | No | No |
| Ga0596861_0007_421437_421754 | Esterase/lipase | No | No |
| Ga0596861_0002_246681_248006 | Esterase/lipase superfamily | Yes | No |
| Ga0596861_0288_154_453 | Predicted esterase | No | No |
| Ga0596861_0018_119595_120236 | Predicted esterase | No | No |
| Ga0596861_0014_6579_8309 | Putative esterase | No | No |
| Ga0596861_0004_456229_458664 | Erythromycin esterase-like protein | No | No |
| Ga0596861_0001_584142_585152 | L-aminopeptidase / D-esterase-like | No | No |
| Ga0596861_0010_206892_207701 | Putative tributyrin esterase | No | No |
| Ga0596861_0436_3_395 | Carboxylesterase type B | No | No |
| Ga0596861_0014_122086_122988 | Phospholipase A1/A2 | No | No |
| Ga0596861_0002_863391_864032 | Acyl-CoA thioesterase I | Yes | No |
| Ga0596861_0005_25679_26659 | Lysophospholipase | No | No |
| Ga0596861_0017_56300_57040 | Acyl-CoA thioesterase I | No | No |
| Ga0596861_0009_371883_372950 | Two-component system, chemotaxis family, protein-glutamate methylesterase/glutaminase | No | No |

**Table S3:** Predicted lipases and esterases identified in UHH-Hm9b genome

| Locus Tag | Gene Product | Signal IP | Transmembrane Helices |
| --- | --- | --- | --- |
| Ga0596863_001_95000_95845 | Acetyl esterase / lipase | No | No |
| Ga0596863_001_96789_97727 | Lysophospholipase L1-like esterase | No | No |
| Ga0596863_001_322982_323869 | Acetyl esterase/lipase | No | No |
| Ga0596863_002_56645_57349 | Esterase/lipase superfamily enzyme | No | No |
| Ga0596863_002_85062_86849 | Lysophospholipase L1-like esterase | No | No |
| Ga0596863_002_227312_228256 | Acetyl esterase/lipase | No | Yes |
| Ga0596863_002_228295_229014 | Lysophospholipase L1-like esterase | No | No |
| Ga0596863_002_259471_260385 | Acetyl esterase/lipase | Yes | No |
| Ga0596863_002_290394_292529 | Pimeloyl-ACP carboxylesterase / lysophospholipase | Yes | No |
| Ga0596863_004_204990_205676 | Lysophospholipase L1-like esterase | Yes | No |
| Ga0596863_004_211073_211732 | Phospholipase/carboxylesterase | No | No |
| Ga0596863_005_152557_153375 | Acetyl esterase/lipase | Yes | No |
| Ga0596863_005_221343_222188 | Acetyl esterase/lipase | No | No |
| Ga0596863_006_50226_51491 | (4-O-methyl)-D-glucuronate esterase | Yes | No |
| Ga0596863_006_92950_94923 | Sialate O-acetylesterase | Yes | No |
| Ga0596863_010_132661_133428 | Esterase | No | No |
| Ga0596863_011_97373_98173 | Putative tributyrin esterase | No | No |
| Ga0596863_012_9622_10449 | Sialic acid acetyl esterase | No | No |
| Ga0596863_012_54963_55610 | Predicted esterase | No | No |
| Ga0596863_015_57851_58804 | Acetyl esterase/lipase | No | No |
| Ga0596863_015_58801_59757 | Acetyl esterase/lipase | No | No |
| Ga0596863_023_12895_14946 | Cephalosporin-C deacetylase–like acetyl esterase | No | No |
| Ga0596863_036_9578_10585 | Esterase/lipase | No | Yes |
| Ga0596863_041_6584_9937 | Lignin esterase | No | No |
| Ga0596863_045_13729_15132 | Sialate O-acetylesterase | Yes | Yes |
| Ga0596863_194_3_263 | Predicted α/β hydrolase family esterase | No | No |
| Ga0596863_002_249346_250002 | Dienelactone hydrolase | No | No |
| Ga0596863_005_106435_107109 | Dienelactone hydrolase | No | No |
| Ga0596863_010_145617_146798 | Dienelactone hydrolase | No | No |
| Ga0596863_032_11264_13447 | Dienelactone hydrolase | No | Yes |
| Ga0596863_032_71734_73050 | Dienelactone hydrolase | No | No |
| Ga0596863_002_48494_49318 | Predicted alpha/beta superfamily hydrolase | No | No |
| Ga0596863_007_60346_61488 | Predicted alpha/beta superfamily hydrolase | Yes | No |
| Ga0596863_194_3_263 | Predicted alpha/beta hydrolase family esterase | No | No |
| Ga0596863_005_10762_11607 | Alpha-beta hydrolase superfamily lysophospholipase | No | No |
| Ga0596863_007_124767_125372 | 3-keto-disaccharide hydrolase | Yes | No |
| Ga0596863_002_56645_57349 | Esterase/lipase superfamily enzyme | No | No |
| Ga0596863_036_9578_10585 | Esterase/lipase | No | Yes |
| Ga0596863_001_95000_95845 | Acetyl esterase/lipase | No | No |
| Ga0596863_001_322982_323869 | Acetyl esterase/lipase | No | No |
| Ga0596863_002_227312_228256 | Acetyl esterase/lipase | No | Yes |
| Ga0596863_002_259471_260385 | Acetyl esterase/lipase | Yes | No |

**Table S4:** Proteome data set of In UHH-5R5 and UHH-Hm9b grown under planktonic and biofilm conditions (Excel-File)

**Table S5:** Bacterial strains and plasmids used in this study.

| **Strain** | **Phenotype** | **Reference/source** |
| --- | --- | --- |
| *E. coli* DH5α | *supE44 ΔlacU169 (Ф80 lacZ ΔM15) hsdR17 recA1 endA1 gyrA96 thi-1 relA1* | Thermo-Fischer, Germany |
| *E. coli* BL21 (DE3) | *F-, ompT, hsdS B (rB- m B-) gal, dcm, λDE3* | Novagen/Merck, Darmstadt, Germany |
| *E. coli* WM3064 | *thrB1004 pro thi rpsL hsdS lacZΔM15 RP4-1360Δ(araBAD)567 ΔdapA1341::[erm pir(wt)]* | W. Metcalf, University of Illinois, Urbana-Champaign, USA |
| *ReporTPA_UHH04* | *C. thiooxidans S23_UHH01, carrying pReporTPA, Cm^R^* | (27) |
| *Maribacter dokdonensis*  UHH-5R5 | Wild-type strain | This work |
| *Arenibacter palladensis*  UHH-Hm9b | Wild-type strain | This work |
| **Plasmids** | **Characteristics** | **Reference/source** |
| pET21a(+) | Expression vector, *lacI,* Amp^R^, T7-promotor, C-terminal His­_6_-tag coding sequence | Novagen/Merck, Darmstadt, Germany |
| pET21a(+)::PET93 | 1308 bp insert in pET21a(+) coding for PET93 | This work |
| pET21a(+)::PET94 | 1309 bp insert in pET21a(+) coding for PET94 | This work |
|  |  |  |

**Table S6:** Homologs of PET93 and PET94 hydrolases from Bacteroidota isolates UHH-5R5 and UHH-Hm9b. Data were retrieved from the publicly available IMG/MER database, applying thresholds of ≥50% sequence identity and ≥80% sequence coverage.

| Gene ID | | Locus Tag | | | Genome name | | Isolation Country | | Latitude | | Longitude | |  |
| --- | --- | --- | --- | --- | --- | --- | --- | --- | --- | --- | --- | --- | --- |
| UHH-5R5 homologs | | | | | | | | | | | | |  |
| 2546243389 | | ADICYQ_2709 | | | *Cyclobacterium qasimii* M12-11B | | Norway | | 79.00105 | | 11.66625 | |  |
| 2890793592 | | Ga0443375_01_526053_527357 | | | *Cyclobacterium qasimii* NBRC 106168 | | Norway | | 79.00105 | | 11.66625 | |  |
| 2515733714 | | B156DRAFT_01776 | | | *Spirosoma luteum* DSM 19990 | | Norway | | 78.2122 | | 15.8 | |  |
| 2515842746 | | B157DRAFT_06708 | | | *Spirosoma spitsbergense* DSM 19989 | | Norway | | 78.21 | | 15.8 | |  |
| 2774668436 | | Ga0226569_114770 | | | *Arenibacter algicola* SMS7 | | Sweden | | 58.8734 | | 11.07782 | |  |
| 2595693039 | | LX87DRAFT_00794 | | | *Larkinella arboricola* DSM 21851 | | Russia | | 55.75 | | 37.62 | |  |
| 2585370157 | | FG20DRAFT_3328 | | | *Zobellia amurskyensis* MAR_2009_138 | | Germany | | 55.0255 | | 8.4567 | |  |
| 2592972043 | | GQ41DRAFT_0959 | | | *Arenibacter algicola* MAR_2009_79 | | Germany | | 55.0255 | | 8.4567 | |  |
| 2676739754 | | Ga0040979_3239 | | | *Maribacter sp.* MAR_2009_60 | | Germany | | 55.0255 | | 8.4567 | |  |
| 2667678916 | | Ga0040978_1313 | | | *Maribacter dokdonensis* MAR_2009_71 | | Germany | | 55.0255 | | 8.4567 | |  |
| 2832368994 | | Ga0335984_1474 | | | *Zobellia galactanivorans* OII3 | | Germany | | 54.67 | | 9.94 | |  |
| 2574425426 | | P177DRAFT_00341 | | | *Maribacter forsetii* DSM 18668 | | Germany | | 54.1841 | | 7.9 | |  |
| 2558431147 | | P178DRAFT_1169 | | | *Maribacter sp.* Hel_I _7 | | Germany | | 54.1841 | | 7.9 | |  |
| 8000209034 | | Ga0596861_0008_300124_301428 | | | *Maribacter dokdonensis* AG-HH-5R5 | | Germany | | 54.1318 | | 8.8753 | |  |
| 2515427593 | | B036DRAFT_03034 | | | *Neolewinella persica* DSM 23188 | | Ireland | | 53.27 | | -9.056 | |  |
| 2504771602 | | Halhy_2235 | | | *Haliscomenobacter hydrossis* O, DSM 1100 | | Netherlands | | 51.77 | | 5.53 | |  |
| 8065844926 | | Ga0487514_01_1236377_1237705 | | | *Zobellia roscoffensis* Asnod2-B02-B | | France | | 48.7266 | | -3.9897 | |  |
| 3001348201 | | Ga0487513_01_1242554_1243882 | | | *Zobellia nedashkovskayae* Asnod2-B07-B | | France | | 48.7266 | | -3.9897 | |  |
| 8065850772 | | Ga0487512_01_3324766_3326094 | | | *Zobellia nedashkovskayae* Asnod3-E08-A | | France | | 48.7266 | | -3.9897 | |  |
| 3001333670 | | Ga0488450_01_1260666_1261994 | | | *Zobellia roscoffensis* Asnod1-F08 | | France | | 48.7266 | | -3.9897 | |  |
| 2881099053 | | Ga0442556_33_349623_350918 | | | *Arundinibacter roseus* DMA-k-7a | | Hungary | | 47.7 | | 16.69997 | |  |
| 8055965970 | | Ga0616553_11_1042874_1044202 | | | *Zobellia barbeyronii* KMM 6746 | | Russia | | 47.51669 | | 152.813 | |  |
| 648160996 | | FB2170_02060 | | | *Maribacter sp.* HTCC2170 | | USA | | 44.655 | | -124.064 | |  |
| 644104643 | | Flav2ADRAFT_1526 | | | *Flavobacteria bacterium* MS024-2A | | USA | | 43.84978 | | -69.6266 | |  |
| 8080698659 | | Ga0625472_07_32683_33957 | | | *Portibacter lacus* YM8-076 | | Japan | | 43.27284 | | 142.5722 | |  |
| 2910289591 | | Ga0477856_036_38108_39436 | | | *Zobellia amurskyensis* KMM 3526 | | Russia | | 43.14794 | | 131.891 | |  |
| 2890770580 | | Ga0441612_223_154750_156033 | | | *Larkinella sp.* C7 | | China | | 43.1 | | 119.37 | |  |
| 8058070572 | | Ga0617454_01_1769634_1770962 | | | *Zobellia alginiliquefaciens* LLG6346-3.1 | | France | | 42.76904 | | 9.33353 | |  |
| 2919024960 | | Ga0454494_10_5124_6437 | | | *Arcicella sp.* BE51 | | USA | | 42.444 | | -76.5019 | |  |
| 2919355278 | | Ga0454526_11_5124_6437 | | | *Arcicella sp.* BE140 | | USA | | 42.444 | | -76.5019 | |  |
| 2919350065 | | Ga0454525_11_5124_6437 | | | *Arcicella sp.* BE139 | | USA | | 42.444 | | -76.5019 | |  |
| 8048528387 | | Ga0616067_01_1951018_1952100 | | | *Zobellia laminariae* AS94 | | USA | | 42.42 | | -70.907 | |  |
| 3001341769 | | Ga0584015_08_147227_148549 | | | *Zobellia uliginosa* C3R17 | | USA | | 42.25 | | -70.54 | |  |
| 8074547076 | | Ga0580702_01_922024_923352 | | | *Zobellia sp.* B3R18 | | USA | | 42.25 | | -70.54 | |  |
| 8100324962 | | Ga0488656_01_1350439_1351743 | | | *Maribacter sp.* 6B07 | | USA | | 42.25 | | -70.54 | |  |
| 3001904140 | | Ga0580690_04_133467_134774 | | | *Arenibacter algicola* E3M18 | | USA | | 42.25 | | -70.54 | |  |
| 8074614719 | | Ga0580712_45_213521_214849 | | | *Zobellia galactanivorans* A2M03 | | USA | | 42.25 | | -70.54 | |  |
| 2890828893 | | Ga0441110_04_6487_7779 | | | *Runella sp.* CRIBMP | | USA | | 42.23591 | | -81.7859 | |  |
| 2890197314 | | Ga0440108_02_155726_157030 | | | *Emticicia sp.* CRIBPO | | USA | | 42.23591 | | -81.7859 | |  |
| 2808571772 | | Ga0321368_117257 | | | *Spongiimicrobium salis* Da_B9 | | Spain | | 42.11444 | | 3.168333 | |  |
| 2721515523 | | Ga0175219_111860 | | | *Maribacter sp.* 1_2014MBL_MicDiv | | USA | | 41.52733 | | -70.6757 | |  |
| 2884363254 | | Ga0442147_16_52030_53331 | | | *Spirosoma sp.* 209 | | USA | | 40.70124 | | -74.0287 | |  |
| 2890750500 | | Ga0441869_11_349676_350959 | | | *Cyclobacterium roseum* SYSU L10180 | | China | | 40.50182 | | 85.02692 | |  |
| 2890710313 | | Ga0442907_13_418729_420021 | | | *Cyclobacterium sp.* SYSU L10401 | | China | | 40.50182 | | 85.02692 | |  |
| 2890760553 | | Ga0440852_11_379535_380818 | | | *Cyclobacterium sp.* SYSU L10167 | | China | | 40.50182 | | 85.02692 | |  |
| 646858067 | | Trad_2647 | | | *Truepera radiovictrix* RQ-24, DSM 17093 | | Portugal | | 37.946 | | -25.49 | |  |
| 2884494525 | | Ga0441408_01_1388066_1389361 | | | *Maribacter algicola* PoM-212 | | South Korea | | 37.64039 | | 126.5886 | |  |
| 8001640529 | | Ga0594633_08_15931_17229 | | | *Flagellimonas sp.* 389 | | China | | 37.55817 | | 122.0907 | |  |
| 8077179044 | | Ga0627981_45_82466_83773 | | | *Muriicola sp.* Z0-33 | | China | | 37.5292 | | 122.0108 | |  |
| 8099929817 | | Ga0626244_078_5418_6725 | | | *Arenibacter sp.* S6351L | | China | | 37.51718 | | 122.1552 | |  |
| 8077281885 | | Ga0626238_127_136883_138184 | | | *Arenibacter sp.* F20364 | | China | | 37.51118 | | 122.1439 | |  |
| 2897639915 | | Ga0441610_12_1042881_1044170 | | | *Maribacter sp.* RZ26 | | China | | 37.51118 | | 122.1439 | |  |
| 8078693221 | | Ga0627007_04_12753_14045 | | | *Lacihabitans sp.* CCS-44 | | South Korea | | 37.45573 | | 129.1895 | |  |
| 640617222 | | ALPR1_05630 | | | *Algoriphagus machipongonensis* PR1 | | USA | | 37.42 | | -75.69 | |  |
| 8071170371 | | Ga0581854_08_68151_69446 | | | *Spirosoma sp.* RHs26 | | South Korea | | 37.37771 | | 127.5979 | |  |
| 2890815525 | | Ga0440155_36_295790_297082 | | | *Cyclobacterium plantarum* GBPx2 | | Iran | | 37.27387 | | 54.14993 | |  |
| 2623579142 | | SB49_04750 | | | *Sediminicola sp.* YIK13 | | South Korea | | 37.26667 | | 126.4333 | |  |
| 2517148360 | | RudluDRAFT_0983 | | | *Rudanella lutea* DSM 19387 | | South Korea | | 37.263 | | 127.028 | |  |
| 2623582104 | | AAY42_03745 | | | *Muricauda eckloniae* DOKDO 007 | | South Korea | | 37.23333 | | 131.8667 | |  |
| 2861801624 | | Ga0436359_050_168825_170123 | | | *Cyclobacterium marinum* Atlantic-IS | | North Atlantic Ocean | | 37.0567 | | -73.5113 | |  |
| 2845821377 | | Ga0398781_01_3824902_3826197 | | | *Flavobacteriaceae bacterium* F202Z8 | | South Korea | | 36.78109 | | 126.2954 | |  |
| 2834236294 | | Ga0337323_3415 | | | *Maribacter litoralis* SDRB-Phe2 | | South Korea | | 36.66617 | | 126.2106 | |  |
| 2906824236 | | Ga0446167_12_10442_11746 | | | *Flavobacterium sp.* ASW18X | | China | | 36.44574 | | 120.798 | |  |
| 2920457388 | | Ga0481285_24_11737_13044 | | | *Arenibacter arenosicollis* BSSL-BM3 | | South Korea | | 36.37273 | | 126.586 | |  |
| 2967498004 | | Ga0440713_44_196380_197681 | | | *Lunatibacter salilacus* CUG 91308 | | China | | 36.33 | | 100.37 | |  |
| 2920068231 | | Ga0477952_12_16130_17458 | | | *Limnovirga soli* KCS-6 | | South Korea | | 36.3 | | 127.3 | |  |
| 2832967971 | | Ga0393098_3166 | | | *Muricauda aurantiaca* HME9304 | | South Korea | | 36.0759 | | 120.4085 | |  |
| 2890885683 | | Ga0440596_07_627843_629129 | | | *Flagellimonas algicola* AsT0115 | | South Korea | | 36 | | 126 | |  |
| 8055114222 | | Ga0616906_03_99341_100636 | | | *Spirosoma liriopis* RP8 | | South Korea | | 35.9078 | | 127.7669 | |  |
| 2774555809 | | Ga0198768_112759 | | | *Deinococcus puniceus* DY1 | | South Korea | | 35.86056 | | 127.7464 | |  |
| 2571070915 | | Q371DRAFT_04942 | | | *Deinococcus misasensis* DSM 22328 | | Japan | | 35.41927 | | 133.8642 | |  |
| 2790839146 | | Ga0304276_1007138 | | | *Maribacter sp.* 4G9 | | Japan | | 35.16 | | 139.61 | |  |
| 3000161165 | | Ga0442852_02_374859_376166 | | | *Cytophagaceae bacterium* SJW1-29 | | South Korea | | 35.08978 | | 127.7449 | |  |
| 8078051908 | | Ga0626623_09_414643_415917 | | | *Flagellimonas sp.* 2012CJ39-3 | | South Korea | | 33.3846 | | 126.5535 | |  |
| 8064342266 | | Ga0559750_01_4033798_4035096 | | | *Flagellimonas sp.* CMM7 | | South Korea | | 33 | | 126 | |  |
| 2890835923 | | Ga0444202_07_441737_443035 | | | *Flagellimonas sp.* CMM7 | | South Korea | | 33 | | 126 | |  |
| 2509080589 | | DeipiDRAFT_00001900 | | | *Deinococcus pimensis* KR-235, DSM 21231 | | USA | | 32.92784 | | -112.305 | |  |
| 8002456163 | | Ga0594566_01_865258_866562 | | | *Maribacter sp.* MMG018 | | USA | | 32.73299 | | -117.257 | |  |
| 8124090958 | | Ga0674574_01_5119903_5121201 | | | *Imperialibacter roseus* P4T | | USA | | 31.2714 | | -102.68 | |  |
| 8026246356 | | Ga0444592_04_28376_29677 | | | *Bacteroidetes bacterium* AG-901-E05 | | Atlantic Ocean | | 31.07 | | -64.17 | |  |
| 8026765874 | | Ga0447146_12_39013_40299 | | | *Bacteroidetes bacterium* AG-892-D16 | | Atlantic Ocean | | 31.07 | | -64.17 | |  |
| 2505793149 | | Runsl_3883 | | | *Runella slithyformis* LSU4, DSM 19594 | | USA | | 30.417 | | -91.167 | |  |
| 2832454624 | | Ga0337407_5034 | | | *Runella aurantiaca* YX9 | | China | | 27.96722 | | 112.8431 | |  |
| 2506486384 | | Emtol_0927 | | | *Emticicia oligotrophica* GPTSA100-15, DSM 17448 | | India | | 26.75602 | | 94.20945 | |  |
| 2832106458 | | Ga0336645_3811 | | | *Fibrisoma montanum* HYT19 | | China | | 25.04244 | | 113.7427 | |  |
| 2914067816 | | Ga0442815_14_714997_716286 | | | *Pareuzebyella sediminis* S2-4-21 | | China | | 24.85 | | 118.67 | |  |
| 2964275936 | | Ga0442816_07_673424_674713 | | | *Pareuzebyella sediminis* MT2-5-19 | | China | | 24.85 | | 118.67 | |  |
| 8046923320 | | Ga0615507_11_371971_373236 | | | *Portibacter marinus* 10MBP4-2-1 | | China | | 24.81823 | | 118.6732 | |  |
| 2700893107 | | Ga0137942_100379 | | | *Cytophaga sp.* FL35 | | USA | | 24.56306 | | -81.4008 | |  |
| 8063583800 | | Ga0607407_03_90615_91904 | | | *Cytophaga sp.* FL35 | | USA | | 24.56306 | | -81.4008 | |  |
| 2723494309 | | Ga0175042_111868 | | | *Maribacter hydrothermalis* T28 | | Taiwan | | 24 | | 121 | |  |
| 2994701013 | | Ga0587408_078_152776_154110 | | | *Lunatimonas salinarum* KCTC 42988 | | India | | 21.85524 | | 72.32298 | |  |
| 2548890299 | | GCKDRAFT_01856 | | | *Croceivirga radicis* S86 | | Micronesia | | 11.523 | | 151.494 | |  |
| 2982560847 | | Ga0577150_01_1908993_1910297 | | | *Maribacter sp.* 151 | | Australia | | -38.3336 | | 142.621 | |  |
| 8002498218 | | Ga0594204_44_82137_83441 | | | *Algoriphagus aquimarinus* ACAM 450 | | Antarctica | | -68 | | 78 | |  |
| 2894866264 | | Ga0441611_037_63082_64380 | | | *Maribacter sp.* ACAM166 | | Antarctica | | -68 | | 78 | |  |
| 2792181821 | | Ga0309199_12455 | | | *Arenibacter catalasegens* P308H10 | | Antarctica | | -69 | | 76 | |  |
| 2739624306 | | Ga0196854_10569 | | | *Algoriphagus antarcticus* DSM 15986 (v2) (version 2) | | Antarctica | | -69.385 | | 76.37841 | |  |
| 8002500727 | | Ga0559560_049_173548_174849 | | | *Algoriphagus antarcticus* DSM 15986 | | Antarctica | | -69.385 | | 76.37841 | |  |
| Hm9b homologs | | | | | | | | | | | |  | |
| 2546243389 | ADICYQ_2709 | | | *Cyclobacterium qasimii* M12-11B | | | Norway | 79.00105 | | 11.66625 | |  | |
| 2890793592 | Ga0443375_01_526053_527357 | | | *Cyclobacterium qasimii* NBRC 106168 | | | Norway | 79.00105 | | 11.66625 | |  | |
| 2515733714 | B156DRAFT_01776 | | *Spirosoma luteum* DSM 19990 | | | Norway | | 78.2122 | | 15.8 | |  | |
| 2515842746 | B157DRAFT_06708 | | *Spirosoma spitsbergense* DSM 19989 | | | Norway | | 78.21 | | 15.8 | |  | |
| 2774668436 | Ga0226569_114770 | | *Arenibacter algicola* SMS7 | | | Sweden | | 58.87242 | | 11.06366 | |  | |
| 2778165481 | Ga0248407_161130 | | *Janthinobacterium psychrotolerans* S3-2 | | | Denmark | | 56.1828 | | 10.17629 | |  | |
| 2595693039 | LX87DRAFT_00794 | | *Larkinella arboricola* DSM 21851 | | | Russia | | 55.75 | | 37.62 | |  | |
| 2667678916 | Ga0040978_1313 | | *Maribacter dokdonensis* MAR_2009_71 | | | Germany | | 55.0255 | | 8.4567 | |  | |
| 2585370157 | FG20DRAFT_3328 | | *Zobellia amurskyensis* MAR_2009_138 | | | Germany | | 55.0255 | | 8.4567 | |  | |
| 2592972043 | GQ41DRAFT_0959 | | *Arenibacter algicola* MAR_2009_79 | | | Germany | | 55.0255 | | 8.4567 | |  | |
| 2676739754 | Ga0040979_3239 | | *Maribacter sp.* MAR_2009_60 | | | Germany | | 55.0255 | | 8.4567 | |  | |
| 2832368994 | Ga0335984_1474 | | *Zobellia galactanivorans* OII3 | | | Germany | | 54.67 | | 9.94 | |  | |
| 2558431147 | P178DRAFT_1169 | | *Maribacter sp.* Hel_I _7 | | | Germany | | 54.1841 | | 7.9 | |  | |
| 2574425426 | P177DRAFT_00341 | | *Maribacter forsetii* DSM 18668 | | | Germany | | 54.1841 | | 7.9 | |  | |
| 8000209034 | Ga0596861_0008_300124_301428 | | *Maribacter dokdonensis* AG-HH-5R5 | | | Germany | | 54.1318 | | 8.8753 | |  | |
| 2515427593 | B036DRAFT_03034 | | *Neolewinella persica* DSM 23188 | | | Ireland | | 53.27 | | -9.056 | |  | |
| 8001908943 | Ga0594730_11_249862_251190 | | *Zobellia russellii* KMM 3677 | | | Russia | | 52.33419 | | 36.60303 | |  | |
| 2910278776 | Ga0477857_08_1217993_1219321 | | *Zobellia laminariae* KMM 3676 | | | Russia | | 52.33419 | | 36.60303 | |  | |
| 8065844926 | Ga0487514_01_1236377_1237705 | | *Zobellia roscoffensis* Asnod2-B02-B | | | France | | 48.7266 | | -3.9897 | |  | |
| 3001333670 | Ga0488450_01_1260666_1261994 | | *Zobellia roscoffensis* Asnod1-F08 | | | France | | 48.7266 | | -3.9897 | |  | |
| 3001348201 | Ga0487513_01_1242554_1243882 | | *Zobellia nedashkovskayae* Asnod2-B07-B | | | France | | 48.7266 | | -3.9897 | |  | |
| 8065850772 | Ga0487512_01_3324766_3326094 | | *Zobellia nedashkovskayae* Asnod3-E08-A | | | France | | 48.7266 | | -3.9897 | |  | |
| 2620655879 | Ga0039355_111800 | | *Zobellia galactanivorans* DsijT | | | France | | 48.14524 | | -4.35629 | |  | |
| 2881099053 | Ga0442556_33_349623_350918 | | *Arundinibacter roseus* DMA-k-7a | | | Hungary | | 47.7 | | 16.69997 | |  | |
| 8055965970 | Ga0616553_11_1042874_1044202 | | *Zobellia barbeyronii* KMM 6746 | | | Russia | | 47.52363 | | 152.8107 | |  | |
| 648160996 | FB2170_02060 | | *Maribacter sp.* HTCC2170 | | | USA | | 44.655 | | -124.064 | |  | |
| 644104643 | Flav2ADRAFT_1526 | | *Flavobacteria bacterium* MS024-2A (unscreened) | | | USA | | 43.84978 | | -69.6266 | |  | |
| 8080698659 | Ga0625472_07_32683_33957 | | *Portibacter lacus* YM8-076 | | | Japan | | 43.20081 | | 142.5392 | |  | |
| 2910289591 | Ga0477856_036_38108_39436 | | *Zobellia amurskyensis* KMM 3526 | | | Russia | | 43.13641 | | 131.8848 | |  | |
| 2890770580 | Ga0441612_223_154750_156033 | | *Larkinella sp.* C7 | | | China | | 43.1 | | 119.37 | |  | |
| 8058070572 | Ga0617454_01_1769634_1770962 | | *Zobellia alginiliquefaciens* LLG6346-3.1 | | | France | | 42.76904 | | 9.33353 | |  | |
| 8048528387 | Ga0616067_01_1951018_1952100 | | *Zobellia laminariae* AS94 | | | USA | | 42.42 | | -70.907 | |  | |
| 3001341769 | Ga0584015_08_147227_148549 | | *Zobellia uliginosa* C3R17 | | | USA | | 42.25 | | -70.54 | |  | |
| 8074547076 | Ga0580702_01_922024_923352 | | *Zobellia sp.* B3R18 | | | USA | | 42.25 | | -70.54 | |  | |
| 3001904140 | Ga0580690_04_133467_134774 | | *Arenibacter algicola* E3M18 | | | USA | | 42.25 | | -70.54 | |  | |
| 8100324962 | Ga0488656_01_1350439_1351743 | | *Maribacter sp.* 6B07 | | | USA | | 42.25 | | -70.54 | |  | |
| 8074614719 | Ga0580712_45_213521_214849 | | *Zobellia galactanivorans* A2M03 | | | USA | | 42.25 | | -70.54 | |  | |
| 2890197314 | Ga0440108_02_155726_157030 | | *Emticicia sp.* CRIBPO | | | USA | | 42.23591 | | -81.7859 | |  | |
| 2890828893 | Ga0441110_04_6487_7779 | | *Runella sp.* CRIBMP | | | USA | | 42.23591 | | -81.7859 | |  | |
| 2808571772 | Ga0321368_117257 | | *Spongiimicrobium salis* Da_B9 | | | Spain | | 42.11444 | | 3.168333 | |  | |
| 2721515523 | Ga0175219_111860 | | *Maribacter sp.* 1_2014MBL_MicDiv | | | USA | | 41.52733 | | -70.6757 | |  | |
| 2890760553 | Ga0440852_11_379535_380818 | | *Cyclobacterium sp.* SYSU L10167 | | | China | | 40.50182 | | 85.02692 | |  | |
| 2890750500 | Ga0441869_11_349676_350959 | | *Cyclobacterium roseum* SYSU L10180 | | | China | | 40.4684 | | 85.51032 | |  | |
| 2515907397 | B153DRAFT_02476 | | *Spirosoma panaciterrae* DSM 21099 | | | South Korea | | 37.91626 | | 127.2038 | |  | |
| 2516421498 | B154DRAFT_00033 | | *Segetibacter koreensis* DSM 18137 | | | South Korea | | 37.91626 | | 127.2038 | |  | |
| 2700210779 | Ga0131097_3515 | | *Algoriphagus halophilus* DSM 15292 | | | South Korea | | 37.71317 | | 126.45 | |  | |
| 2897639915 | Ga0441610_12_1042881_1044170 | | *Maribacter sp.* RZ26 | | | China | | 37.55838 | | 122.1533 | |  | |
| 8077179044 | Ga0627981_45_82466_83773 | | *Muriicola sp.* Z0-33 | | | China | | 37.5292 | | 122.0108 | |  | |
| 8078693221 | Ga0627007_04_12753_14045 | | *Lacihabitans sp.* CCS-44 | | | South Korea | | 37.45246 | | 129.1922 | |  | |
| 640617222 | ALPR1_05630 | | *Algoriphagus machipongonensis* PR1 | | | USA | | 37.42 | | -75.69 | |  | |
| 8071170371 | Ga0581854_08_68151_69446 | | *Spirosoma sp.* RHs26 | | | South Korea | | 37.38044 | | 127.601 | |  | |
| 2890815525 | Ga0440155_36_295790_297082 | | *Cyclobacterium plantarum* GBPx2 | | | Iran | | 37.28586 | | 54.12379 | |  | |
| 2623579142 | SB49_04750 | | *Sediminicola sp.* YIK13 | | | South Korea | | 37.26667 | | 126.4333 | |  | |
| 2517148360 | RudluDRAFT_0983 | | *Rudanella lutea* DSM 19387 | | | South Korea | | 37.263 | | 127.028 | |  | |
| 2623582104 | AAY42_03745 | | *Muricauda eckloniae* DOKDO 007 | | | South Korea | | 37.23333 | | 131.8667 | |  | |
| 2861801624 | Ga0436359_050_168825_170123 | | *Cyclobacterium marinum* Atlantic-IS | | | USA | | 37.0567 | | -73.5113 | |  | |
| 2832426529 | Ga0337346_2388 | | *Muricauda koreensis* ECD12 | | | South Korea | | 36.79991 | | 126.4363 | |  | |
| 2506468824 | Cycma_3952 | | *Cyclobacterium marinum* Raj, DSM 745 | | | USA | | 36.67702 | | -120.005 | |  | |
| 2906824236 | Ga0446167_12_10442_11746 | | *Flavobacterium sp.* ASW18X | | | China | | 36.44574 | | 120.798 | |  | |
| 2967498004 | Ga0440713_44_196380_197681 | | *Lunatibacter salilacus* CUG 91308 | | | China | | 36.33 | | 100.37 | |  | |
| 2920068231 | Ga0477952_12_16130_17458 | | *Limnovirga soli* KCS-6 | | | South Korea | | 36.3 | | 127.3 | |  | |
| 2832967971 | Ga0393098_3166 | | *Muricauda aurantiaca* HME9304 | | | South Korea | | 36.0759 | | 120.4085 | |  | |
| 2890885683 | Ga0440596_07_627843_629129 | | *Flagellimonas algicola* AsT0115 | | | South Korea | | 36 | | 126 | |  | |
| 8055114222 | Ga0616906_03_99341_100636 | | *Spirosoma liriopis* RP8 | | | South Korea | | 35.9078 | | 127.7669 | |  | |
| 2790839146 | Ga0304276_1007138 | | *Maribacter sp.* 4G9 | | | Japan | | 35.16 | | 139.61 | |  | |
| 8078690697 | Ga0627006_023_48688_49980 | | *Lacihabitans sp.* CS3-21 | | | South Korea | | 35.04876 | | 128.9176 | |  | |
| 8078051908 | Ga0626623_09_414643_415917 | | *Flagellimonas sp.* 2012CJ39-3 | | | South Korea | | 33.3846 | | 126.5535 | |  | |
| 2889832122 | Ga0443539_12_800808_802112 | | *Croceivirga sp.* JEA036 | | | South Korea | | 33.25849 | | 126.6494 | |  | |
| 8064342266 | Ga0559750_01_4033798_4035096 | | *Flagellimonas sp.* CMM7 | | | South Korea | | 33 | | 126 | |  | |
| 2890835923 | Ga0444202_07_441737_443035 | | *Flagellimonas sp.* CMM7 | | | South Korea | | 33 | | 126 | |  | |
| 8002456163 | Ga0594566_01_865258_866562 | | *Maribacter sp.* MMG018 | | | USA | | 32.73288 | | -117.257 | |  | |
| 8099924753 | Ga0626761_23_337354_338775 | | *Arenibacter sp.* N53 | | | China | | 31.43939 | | 122.2886 | |  | |
| 8124090958 | Ga0674574_01_5119903_5121201 | | *Imperialibacter roseus* P4T | | | USA | | 31.2714 | | -102.68 | |  | |
| 8026765874 | Ga0447146_12_39013_40299 | | *Bacteroidetes bacterium* AG-892-D16 | | | Atlantic Ocean | | 31.07 | | -64.17 | |  | |
| 8026246356 | Ga0444592_04_28376_29677 | | *Bacteroidetes bacterium* AG-901-E05 | | | Atlantic Ocean | | 31.07 | | -64.17 | |  | |
| 2505793149 | Runsl_3883 | | *Runella slithyformis* LSU4, DSM 19594 | | | USA | | 30.417 | | -91.167 | |  | |
| 2789098472 | Ga0303171_1004209 | | *Arsenicibacter rosenii* SM-1 | | | China | | 29.6103 | | 111.3468 | |  | |
| 2832454624 | Ga0337407_5034 | | *Runella aurantiaca* YX9 | | | China | | 27.96722 | | 112.8431 | |  | |
| 2832544890 | Ga0336335_3116 | | *Larkinella punicea* ZZJ9 | | | China | | 27.83097 | | 112.9381 | |  | |
| 2506486384 | Emtol_0927 | | *Emticicia oligotrophica* GPTSA100-15, DSM 17448 | | | India | | 26.75602 | | 94.20945 | |  | |
| 2832106458 | Ga0336645_3811 | | *Fibrisoma montanum* HYT19 | | | China | | 25.04244 | | 113.7427 | |  | |
| 2914067816 | Ga0442815_14_714997_716286 | | *Pareuzebyella sediminis* S2-4-21 | | | China | | 24.85 | | 118.67 | |  | |
| 2964275936 | Ga0442816_07_673424_674713 | | *Pareuzebyella sediminis* MT2-5-19 | | | China | | 24.85 | | 118.67 | |  | |
| 8046923320 | Ga0615507_11_371971_373236 | | *Portibacter marinus* 10MBP4-2-1 | | | China | | 24.81723 | | 118.6649 | |  | |
| 2700893107 | Ga0137942_100379 | | *Cytophaga sp.* FL35 | | | USA | | 24.56306 | | -81.4008 | |  | |
| 8063583800 | Ga0607407_03_90615_91904 | | *Cytophaga sp.* FL35 | | | USA | | 24.56306 | | -81.4008 | |  | |
| 2723494309 | Ga0175042_111868 | | *Maribacter hydrothermalis* T28 | | | Taiwan | | 24 | | 121 | |  | |
| 8002489485 | Ga0594444_49_42594_43904 | | *Algoriphagus pacificus* YJ13C | | | China | | 23.81248 | | 114.4554 | |  | |
| 2994701013 | Ga0587408_078_152776_154110 | | *Lunatimonas salinarum* KCTC 42988 | | | India | | 21.85524 | | 72.32298 | |  | |
| 2789752970 | Ga0303181_101167 | | *Croceivirga radicis* HSG9 | | | China | | 19.7011 | | 109.8361 | |  | |
| 2548890299 | GCKDRAFT_01856 | | *Croceivirga radicis* S86 | | | Micronesia | | 11.523 | | 151.494 | |  | |
| 2881390997 | Ga0440289_056_15317_16636 | | *Flavilitoribacter nigricans* DSM 23189 | | | Nigeria | | 9.494648 | | 7.879082 | |  | |
| 2982560847 | Ga0577150_01_1908993_1910297 | | *Maribacter sp.* 151 | | | Australia | | -38.3336 | | 142.621 | |  | |
| 8002498218 | Ga0594204_44_82137_83441 | | *Algoriphagus aquimarinus* ACAM 450 | | | Antarctica | | -68 | | 78 | |  | |
| 2894866264 | Ga0441611_037_63082_64380 | | *Maribacter sp.* ACAM166 | | | Antarctica | | -68 | | 78 | |  | |
| 2792181821 | Ga0309199_12455 | | *Arenibacter catalasegens* P308H10 | | | Antarctica | | -69 | | 76 | |  | |
| 2739624306 | Ga0196854_10569 | | *Algoriphagus antarcticus* DSM 15986 (v2) (version 2) | | | Antarctica | | -69.385 | | 76.37841 | |  | |
| 8002500727 | Ga0559560_049_173548_174849 | | *Algoriphagus antarcticus* DSM 15986 | | | Antarctica | | -69.385 | | 76.37841 | |  | |
| 8077281885 | Ga0626238_127_136883_138184 | | *Arenibacter sp.* F20364 | | | China | | 37.51118 | | 122.1439 | |  | |
